# A Novel Approach for the Preparation of Tetrathionate Ionic Liquids and Study of Their Sulfur Dissolution Properties

**DOI:** 10.3390/ijms26199823

**Published:** 2025-10-09

**Authors:** Luca Guglielmero, Stefano Becherini, Felicia D’Andrea, Lorenzo Guazzelli, Christian Silvio Pomelli, Alberto Renato de Angelis, Ivan Maffeis, Wallace O’Neil Parker, Andrea Mezzetta

**Affiliations:** 1 Dipartimento di Farmacia, Università di Pisa, Via Bonanno 33, 56126 Pisa, Italy; luca.guglielmero@sns.it (L.G.); s.becherini89@gmail.com (S.B.); felicia.dandrea@unipi.it (F.D.); lorenzo.guazzelli@unipi.it (L.G.); christian.pomelli@unipi.it (C.S.P.); 2 Scuola Normale Superiore, Piazza dei Cavalieri 7, 56126 Pisa, Italy; 3 Eni S.p.A. Research and Technological Innovation Department, via F. Maritano 26, 20097 San Donato Milanese, Italy; alberto.de.angelis@eni.com (A.R.d.A.); ivan.maffeis@eni.com (I.M.);

**Keywords:** sulfur dissolution, tetrathionates, ionic liquids

## Abstract

A series of sulfur dissolving tetrathionate ionic liquids (ILs), featuring imidazolium and pyridinium cationic heads, have been prepared and characterized along with their chloride IL precursors. A novel synthetic approach for the preparation of the proposed tetrathionate ILs has been introduced and successfully tested in the current work, yielding the desired compounds in quantitative yield and high purity, offering a significant advancement over the traditional Volynskii–Smolyaninov reaction. The presented method addresses key challenges of the traditional approach, solving the issues deriving from the influence of the IL cation on the reaction outcome and the unpredictability of the formed polythionate species. The solubility of elemental sulfur in the considered tetrathionate ILs has been investigated at various temperatures, providing good preliminary evidence of the suitability of these ILs as convenient and effective sulfur solubilizing media readily available on the field in “sour” gas extraction plants. Furthermore, the use of ILs instead of traditional organic solvents in operative conditions represents a noteworthy safety improvement due to their lower flammability and volatility. Finally, interesting results were obtained studying binary mixtures of organic solvents and ILs, with cooperative effects or salting-out effects being observed in relation to the type of solvent used.

## 1. Introduction

Elemental sulfur is an inexpensive and abundant resource, which, differently from many other prime materials, is mainly produced as a byproduct of petrochemical and metallurgical industrial processes [1]. Its supply therefore does not follow the common demand-related mechanisms, and its overproduction with respect to the industrial request makes it often considered more likely a waste product than useful material [2,3]. Moreover, increasing global energy needs are leading to the exploitation of sulfur-richer oil and natural gas fields [4], and the desulfurization treatments necessary for the use of these “sour” reserves are expected to further enhance the oversupply of sulfur in the world market [3]. Despite being primarily generated as a byproduct of other commodities, sulfur certainly represents an important, versatile, and valuable output, whose abundance and convenience should be regarded as an opportunity for the development and implementation of more sustainable sulfur-based technologies [1,3]. Besides its most traditional uses, namely, the production of sulfuric acid and the preparation of fertilizers [1,5,6], sulfur is regarded as an interesting material in the context of the green energy transition [7,8,9,10], both for the development of energy electrochemical storage systems [11,12,13,14,15] and realization of thermal storage devices [16,17,18,19,20]. Finally, an intense research activity has been directed toward the study of sulfur as a precursor for the preparation of innovative materials. Among them, it is worth mentioning sulfur-based polymers [21,22], conducting and redox materials with proposed applications in electrochemistry and sensors [23,24,25,26], and sulfur-based building materials, like sulfur concrete or asphalt [27,28,29,30], which also display intriguing recyclability possibilities [31,32].

If it is true that the sulfur produced by the desulfurization of “sour” gas represents a useful resource, the management of “sour” gas reserves pose additional difficulties with respect to “sweeter” sources [1,4,33,34,35]. During the gas expansion, the consequent reduction in pressure and temperature can lead to precipitation of the sulfur compounds dissolved in the gas, with formation of solid sulfur particles. Such a deposition represents a major drawback in the operation of the pipelines and the gas extraction facilities, since elemental sulfur is likely to stock up in pressure control valves, resulting in the stoppage of the stem movement or plugging of the valve orifice [33,36]. The desulfurization of “sour” gas is usually performed by converting the H_2_S in S_8_ [1,5,37,38]. This is typically achieved through a Claus or HydroClaus process, which sees the combustion of part of the H_2_S to SO_2_, used to oxidize the remaining H_2_S to S_8_. Elemental sulfur is not the only product that can be obtained. By carefully setting the ratio between H_2_S and SO_2_, it is indeed possible to steer the reaction toward the production of polythionates of variable lengths, down to thiosulfate [6]. Sulfur and polythionate mixtures have been proved to represent well-performing fertilizers and soil improvers for agricultural purposes, being able to provide plant nutrients in both short and long terms and effective in correcting the alkalinity of soils [6]. Furthermore, polythionates and other sulfur-based salts find metallurgical applications in the chemical milling of magnesium and its alloys as well as in metal machining and gold leaching processes [5,39,40,41,42].

As proposed in a recent work by Spatolisano et al., the preparation of polythionate ionic liquids represents an appealing option for their separation from the aqueous environment at the end of the HydroClaus process, exploiting the low water solubility of this class of compounds [5]. This is achieved by adding an amine or a quaternary ammonium hydroxide salt during the HydroClaus process, which leads to a complete separation of the easily recoverable polythionate ionic liquid and also exhibits catalytic activity toward the reaction between H_2_S and SO_2_. The structure of the amine, in particular, appears to play a key role in the determination of the type of polythionate formed [42,43,44,45,46], with long chained quaternary ammonium bases directing the reaction toward the preferential formation of tetrathionates [5,42,45]. Despite the good potentialities of polythionate systems, at the best of our knowledge, the preparation and study of tetrathionate ionic liquids have been almost completely neglected in the literature up to date, with the only mention of these systems in the work of Spatolisano et al. [5]

In this paper, a set of tetrathionate ionic liquids has been prepared and characterized, following a novel highly performing synthetic strategy. The proposed procedure offers an easier and more generally applicable approach with respect to the traditional one described by Volynskii et al. and by Smolyaninov et al. [43,44,45,47], allowing the preparation of the desired ILs in high purity and removing the influence of the cation type on the outcome of the reaction. Finally, the sulfur dissolution capabilities of the prepared tetrathionate systems and their halide precursors have been tested for pure ILs and in mixtures with organic solvents, providing satisfactory results. The use of tetrathionate and, more generally, polythionate ILs for the dissolution of sulfur is of particular interest. The production of polythionate ILs, as mentioned above, has already been proposed at the industrial level as a way to boost the efficiency of the HydroClaus process [5]. Polythionate ILs can consequently be regarded as highly interesting candidates for the dissolution of sulfur obstructions in sour gas facilities, as they would be readily available solvents derived from the same HydroClaus process taking place in the plant to “sweeten” the extracted gas, further valorizing the “sour” gas desulfurization byproducts. On the other hand, the use of ILs as sulfur-deposit-removal media in the pipelines of “sour” gas extraction facilities appears preferable with respect to traditional organic solvents due their negligible vapor pressure and lower flammability, which makes them a significantly safer and more easily manageable option.

## 2. Results and Discussion

### 2.1. Synthesis

The preparation of tetrathionate (and, more in general, of polythionate) salts is achieved in the literature by adopting the Volynskii–Smolyaninov procedure [47]. This reaction consists of the acidification, at room temperature, of an aqueous solution of sodium thiosulfate, with hydrochloric acid, in the presence of a tetraalkylammonium salt, leading to the formation of a mixture of tetraalkylammonium polythionates and the precipitation of elemental sulfur. The reaction was proposed for the first time in 1963 [47] and, to the best of our knowledge, it still represents the only published method for the preparation of this class of ILs. Despite this reaction having been presented as a good method to prepare polythionate salts, this synthetic pathway suffers considerable limitations, and many experimental parameters need to be carefully calibrated to reach a satisfactory outcome of the procedure. The solution of sodium thiosulfate and tetraalkylammonium salt must be acidified very slowly, and the acid needs to be at the right concentration, otherwise the sodium thiosulfate decomposes immediately, leading to an abundant formation of elemental sulfur and very low yields of the desired polythionate salts. Furthermore, the type of salt used as substrate appears to strongly affect the reaction. No polythionate formation could be observed for tetraalkylammonium salts featuring alkyl chains with less than five carbon atoms, while, depending on the type of ammonium salts employed, tetrathionates, pentathionates, or non-separable mixtures of penta- and tetrathionates salts were obtained [42,43,44,45,46]. Finally, the isolation of the desired products from the reaction mixture is laborious, requiring many organic solvents and multiple purification steps [42]. It appears clear then that the Volynskii–Smolyaninov reaction, despite being capable of providing sufficient results when performed on certain substrate, cannot be regarded as a universally applicable nor a reliable approach for the preparation of tetrathionate ILs.

**Scheme 1:**
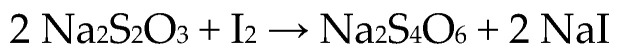
Redox reaction at the base of the iodometric titration system.

To overcome these experimental and structural limitations, the first aim of this study was set as the development of a novel synthetic strategy capable of selectively and efficiently yielding tetrathionate ILs through a simple, quick, and scalable procedure. A two-step process was considered: a first step consisting of the synthesis of sodium tetrathionate, followed by a second step where the prepared sodium tetrathionate is used for the anion metathesis of a halide IL substrate. An efficient and reliable method for the preparation of sodium tetrathionate was provided by the reaction between iodine and sodium thiosulfate (Scheme 1), which is a quantitative and well-known redox process exploited in iodometric titrations [48].

**Scheme 2:**
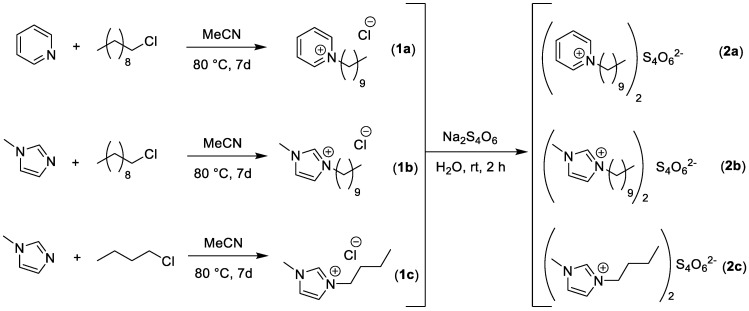
Synthesis of the tetrathionate ILs considered in this study.

A slight excess of iodine was found to be beneficial to reach a full conversion of sodium thiosulfate, and the reaction had to be carried out in a minimal amount of water in order to facilitate the recovery of the highly water-soluble tetrathionate salt. Under these optimized conditions, the desired product could be isolated in excellent yield. The obtained salt was characterized by ATR-FTIR (Figures S1 and S2, Supplementary Materials) and by HRMS, with the obtained data proving the absence of residual thiosulfate, sodium iodide, and other polysulfate species. The performed TGA (Figure S3, Supplementary Materials) exhibited a first mass loss phenomenon at about 100 °C, ascribable to the loss of coordinated water molecules, prior to the main degradation peak observable at higher temperatures. As later described in the Materials and Methods Section, the quantification of this water loss signal allowed for the assessment that the synthesized sodium tetrathionate was recovered as the dihydrate salt Na_2_S_4_O_6_•2H_2_O. The prepared sodium tetrathionate was subsequently employed for the synthesis of three ILs (Scheme 2): *N*-decylpyridinium tetrathionate ([C_10_Py]_2_S_4_O_6_) (**2a**), 1-decyl-3-methylimidazolium tetrathionate ([C_10_MIM]_2_S_4_O_6_) (**2b**) and 1-butyl-3-methylimidazolium tetrathionate ([BMIM]_2_S_4_O_6_) (**2c**). Among these, to the best of our knowledge, [BMIM]_2_S_4_O_6_ and [C_10_MIM]_2_S_4_O_6_ have never been reported in the literature before.

The selected tetrathionate ILs (**2a**–**c**) were obtained in excellent yields through a simple metathesis reaction between a halide IL precursor and a slightly molar excess of sodium tetrathionate (Scheme 2). Tetrathionate ILs display significantly more hydrophobic behavior with respect to their corresponding halide precursor. Compound **2a** was found to exhibit the highest hydrophobicity in the considered set, and during the metathesis reaction, it was already possible to observe the formation of an emulsified double phase composed by water and the forming tetrathionate IL. Nevertheless, both ILs **2a** and **2b** still displayed a water solubility high enough to require their extraction with chloroform to achieve a complete recovery from the aqueous phase. Concerning the preparation of [BMIM]_2_S_4_O_6_, differently from the other long chain (and more hydrophobic) ILs, the chloroform extractions proved to be ineffective due to the much higher solubility of this compound in water. As a result, in this case, it was necessary to perform a more laborious series of precipitations with ethanol (as described in the Materials and Methods Section) in order to recover the desired product. It is worth noting that, differently from the approach reported by Smolyaninov and Volynskii, which was heavily affected by the nature of the substrate, the synthetic pathway proposed in this work enjoys a much wider applicability, overcoming the described limitations [42], and it can be adopted for the preparation of many structurally different ILs.

The obtained ILs were, for the first time, fully characterized by ^1^H and ^13^C NMR, ATR-FTIR and DSC, their viscosity was studied in a range of temperatures, and their performances in the dissolution of elemental sulfur (pure or with a co-solvent) were assessed. Theoretical calculations were finally performed to confirm the formation of sodium tetrathionate and to rationalize the IR characterization of the prepared tetrathionate ILs. HRMS analyses were performed on compounds **2a**–**c**, and, similarly to what was observed for the Na_2_S_4_O_6_•2H_2_O precursor, no residues of halides and no significant presence of polythionates or other byproducts of tetrathionates degradation were observed, indicating that the selected ion metathesis procedure did not affect the stability of the tetrathionate anion.

### 2.2. NMR and ATR-FTIR Characterizations

**Figure 1:**
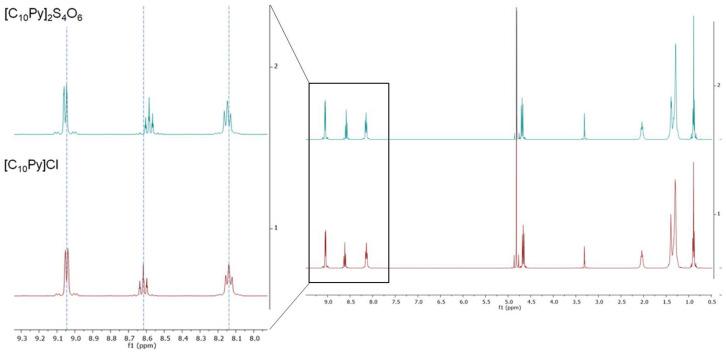
Comparison between ^1^H-NMR of compounds **1a** (bottom) and **2a** (top). A zoom of the 8.0 ppm to 9.3 ppm region is reported on the left.

The prepared ILs were, firstly, characterized by NMR and ATR-FTIR spectroscopies (see Supplementary Materials, Figures S4–S12). While the NMR analyses of the halide ILs **1a**–**c** were consistent with their structure and the literature data [49], in the case of the tetrathionate ILs **2a**–**c**, NMR was not sensitive to the inorganic anion. Nevertheless, a slight shift was observed in the protons and carbons signals of the cationic heads (the imidazolium and pyridinium moieties), which was the molecular region most affected by the interactions with the anions, providing evidence of the change in the paired anion. The observed differences are related both to the different charge density and polarity between chloride and tetrathionate anions, which determine a different interaction with the cationic head protons [50]. On the other hand, no signal shifting was observed for the hydrogens and carbons of alkyl side chains, since their interaction with the anion was usually much less intense. From the comparison in Figure 1, it is possible to appreciate the similarities between the ^1^H-NMR spectra of compounds **1a** and **2a**.

**Figure 2:**
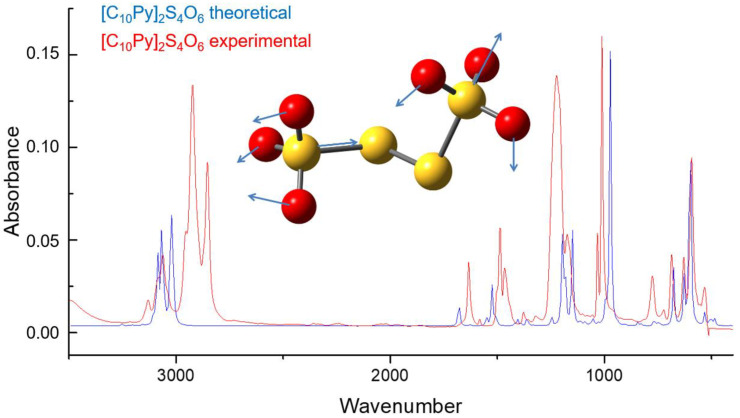
Experimental ATR-FTIR spectrum [C_10_Py]_2_S_4_O_6_ (red) compared with its computational simulation (blue). In the inset figure, the tetrathionate anion is depicted with the counter-phase asymmetric stretching motion normal mode of the two S_2_O_3_ groups (red: oxygen atoms, yellow: sulfur atoms). The blue vectors represent the atom motions. Cations are omitted for clarity.

Different from NMR analysis, FTIR spectroscopy can be directly used to identify the tetrathionate anion. Theoretical calculations have been performed first to simulate the IR spectrum of the tetrathionate anion, in order to assign the peaks observed in the experimental spectrum (Figure S2, Supplementary Materials) with the associated vibrational transition. The vibrational bands observable at 3486 cm^−1^ and 1616 cm^−1^ (Figure S13, Supplementary Materials) are related to the presence of crystallization water molecules (stretching and scissoring, respectively). The broad peak experimentally observed at 1221 cm^−1^ was found to actually result from the superimposition of two signals related to the S=O symmetric stretching of the S_2_O_3_ groups: one associated with the in-phase vibration of the two S_2_O_3_ moieties, the other one associated with their counter-phase vibration (Figure S13, Supplementary Materials). The peak observed at 1027 cm^−1^ and at 1050 cm^−1^ (a shoulder of the 1027 cm^−1^ peak) are associated with the S=O asymmetric stretching (Figure 2). Also in this case, the presence of a set of two coalescent signals is due to the in-phase and counter-phase vibrations of the S_2_O_3_ groups. Finally, the broad band observed at 603 cm^−1^ also emerged to be constituted by a sum of two different signals related to the S-S stretching: a weaker one related to the symmetric S-S stretching, and a stronger one related to the asymmetric S-S stretching (Figure S13, Supplementary Materials).

The couples of in-phase and counter-phase normal mode-related signals, due to the paired motion of the two S_2_O_3_ groups of the S_4_O_6_^2−^, represent an important spectral feature of the tetrathionate system. This pairing is not possible in thiosulphate systems, where there exists a S_2_O_3_ group only, and therefore, this is evidence of the formation of the tetrathionate system. From the comparison between the IR spectra of the tetrathionate ILs with their halide ILs precursors, the emergence of the vibrational bands of the tetrathionate anion is clearly visible (Supplementary Materials, Figures S10–S12). Unexpectedly, the anion bands were found to be more resolved for the ILs than for the tetrathionate sodium salt (the S=O asymmetric stretching and S-S stretching bands, here at 1011 cm^−1^ and 590 cm^−1^, respectively, are better defined and narrow). The IR spectrum of [C_10_Py]_2_S_4_O_6_ (**2a**) was also found to be consistent with the theoretical spectrum obtained by computational means (Figure 2) and with the IR spectrum reported by Spatolisano et al. for the same compound [5].

### 2.3. Thermal and Rheological Characterizations

#### 2.3.1. Differential Scanning Calorimetry (DSC) and Thermal Gravimetric Analysis (TGA)

**Figure 3:**
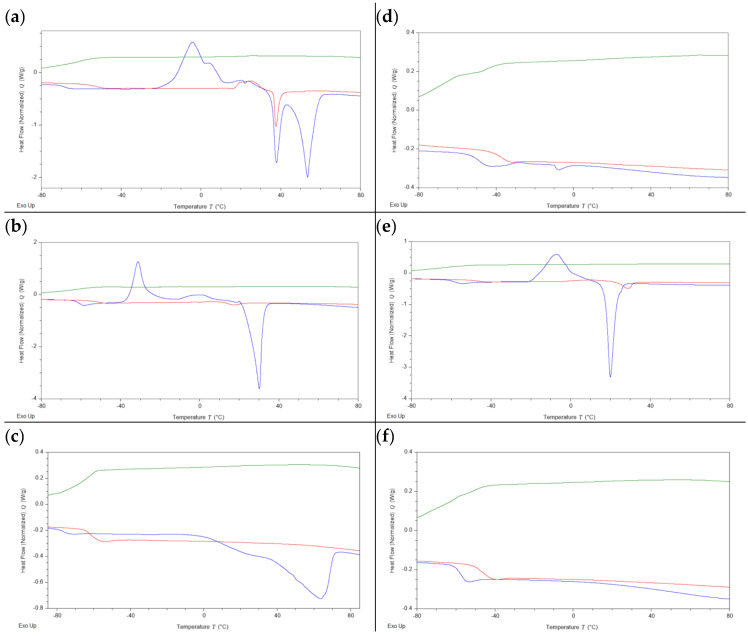
DSC thermograms of the chloride and tetrathionate ILs: (**a**) [C_10_Py]Cl (**1a**), (**b**) [C_10_MIM]Cl (**1b**), (**c**) [BMIM]Cl (**1c**), (**d**) [C_10_Py]_2_S_4_O_6_ (**2a**), (**e**) [C_10_MIM]_2_S_4_O_6_ (**2b**), and (**f**) [BMIM]_2_S_4_O_6_ (**2c**). Blue: first heating run; green: first cooling run; red: second heating run.

The thermal stability of the considered tetrathionate ILs and of sodium tetrathionate, was investigated through thermal gravimetric analysis (TGA), in order to assess the maximum operative temperature of these systems. In addition, the thermal behavior of the synthesized ILs, both featuring the chloride and the tetrathionate anion, was studied by differential scanning calorimetry (DSC). The instrumental procedures that followed are described in the Materials and Methods Section. The recorded TGA thermograms are reported in Supplementary Materials (Figures S3 and S14–S16, Supplementary Materials), while the DSC ones are reported in Figure 3. The obtained DSC and the TGA data are summarized, respectively, in Table 1 and Table 2.

From the obtained TGA results, it is possible to appreciate a clear effect of the cation on thermal stability, even if the recorded difference appeared to be quite mild. The imidazolium ILs displayed the highest thermal stability (*T*_5%_ = 205.84 and 200.87 °C, respectively, for IL **2b** and IL **2c**). The pyridinium IL manifested an intermediate behavior (*T*_5%_ = 196.01 °C), while Na_2_S_4_O_6_ was found to exhibit the lowest thermal stability (*T*_5%_ = 187.31 °C). All the reported thermal stability parameters (*T*_5%_, *T*_onset_ and *T*_peak_) followed the same described trend and manifested similar values among each other. This is a consequence of the very steep first degradation steps exhibited by both Na_2_S_4_O_6_ and ILs **2a**–**c**. Considering the DSC thermograms of the halide ILs **1a**–**c** (Figure 3a–c), it appears evident that the length of the alkyl chain is the key factor for determining their thermal behavior. In the first heating run, long chain ILs **1a** and **1b** displayed a glass transition phenomenon (respectively, at −69.39 °C and −61.70 °C) followed by cold crystallization (respectively, at −4.43 °C and −31.20 °C) and a melting transition (main peaks, respectively, at 53.34 °C and at 30.10 °C). Compound **1a** exhibited polymorphic behavior typical of long alkyl chain cations, and, besides the main thermal events described above, an additional cold crystallization phenomenon associated with the respective fusion event was detectable. In contrast, no polymorphism was observed for compound **1b**. In fact, a single crystallization event associated with melting was observed, and the temperature of this event is in agreement with that reported in the literature [51]. During the second heating run, only the second broad peak of crystallization and the first sharp melting peak were still detectable for **1a**. This means that, during the second heating ramp, the sample was liquid until the endothermic cold crystallization peak at 25.40 °C, followed by its related melting transition at 37.54 °C. In contrast, IL **1b** has a greater tendency to remain an undercooled liquid and only a glass transition at −51.30 °C remained visible during the second heating run. The short chain IL **1c**, solid at room temperature, coherently with the literature data [52], displayed a glass transition, followed by a broader melting phenomenon with respect to what was observed for the long chain ILs.

The type of anion was also found to play, as expected, a major role in the definition of the thermal properties of the studied ILs, with the most evident effects being visible for [C_10_Py]- and [BMIM]-based ILs. Different from the parent IL **1a**, compound **2a** did not display any crystallization or melting transition, and, in both compounds **2a** and **2c**, the observed glass transitions (−51.08 °C and −58.44 °C, respectively) were shifted at higher temperatures with respect to the respective halide IL (Table 1). This effect is reasonable, considering the fact that a divalent anion typically exhibits a higher organizing effect, yielding ionic liquids characterized by stronger interactions among their ions. Curiously, such an evident effect was not observable for [C_10_MIM] systems. In this case the recorded glass transition temperature (−58.91 °C) is quite close to the one observed for the parent compound **1b**, and both the cold crystallization and the melting transitions were retained (peaks −6.91 and 19.96 °C, respectively). Moreover, the melting transition shifted at lower temperatures passing from the monovalent chloride anion to the divalent tetrathionate anion. This peculiar behavior could be attributed to a competition between the organizing effect of the divalent anion and geometrical mismatch, leading to the difficult formation of a stable lattice.

The different thermal behaviors observed between **1a** and **1b** and between **2a** and **2b**, also point out an evident role played by the type of cationic head on the definition of the thermal properties, with the pyridinium head being apparently more affected than the imidazolium one by the transition from the chloride anion to the tetrathionate one.

According to an often-followed literature categorization, the thermal behavior of ILs is divided into three types [53,54]. ILs showing only a glass transition (*T*_g_) in their DSC analysis are defined as belonging to the first type. ILs displaying crystallization (*T*_c_) and melting (*T*_m_) transitions occurring, respectively, during the cooling and the heating run are defined as second-type ILs. Third-type ILs do not crystallize during the cooling run, but during the heating run (cold crystallization (*T*_cc_)), with this signal being followed by a melting event. According to this categorization, compounds **1a**, **1b**, and **2b** are third-type ILs; compound **1c** is a second-type IL; and compounds **2a** and **2c** are first-type ILs.

#### 2.3.2. Viscosity

**Figure 4:**
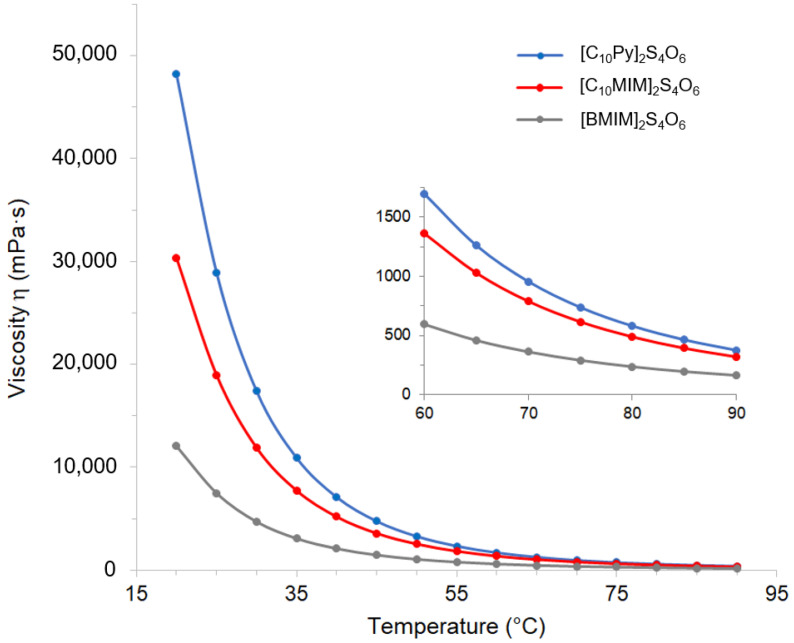
Viscosity of tetrathionate ILs **2a**–**c** as a function of temperature.

Due to strong influence on the mass transport rate, the viscosity of ILs is a central parameter that can be used to define their possible areas of applicability. Often the viscosity of ILs represents one of the main barriers that hinders their use, and usually determines, besides the limit represented by the crystallization temperature, the operative temperature limits in both laboratory and industrial applications. In order to determine the general flow behavior of the synthesized tetrathionate ILs, the viscosity was firstly measured as a function of the shear rate (from 0.1 to 100 s^−1^) at 20 °C, displaying a typical Newtonian behavior for all the ILs in this range. Then, the dependence of the selected ILs viscosity from temperature was evaluated, applying a constant shear rate of 30 s^−1^ and increasing the temperature from 20 °C to 90 °C. For [C_10_Py]Cl, the analyses were performed starting from 40 °C, as it was solid at room temperature. The values obtained are reported in Table S1 and plotted in Figure 4. As is clear from the reported plot, the pyridinium tetrathionate IL exhibited the highest viscosity at 20 °C. As is reported in the literature, the cation-dependent trend in the viscosity is determined by intermolecular interactions. As expected, IL **2a** showed a higher viscosity compared with the related methyl imidazolium tetrathionate IL **2b** (gray line in Figure 4). In fact, the viscosity of ILs with a pyridinium-based cation shows higher viscosity compared with those with an imidazolium-based cation [55]. The length of the side carbon chain is a key variable in the determination of the viscosity properties of an IL. Generally, increasing the length of the side chain causes an increase in the system viscosity, related to the increasing of Van der Waals interactions [56,57,58,59]. The higher viscosity observed for [C_10_MIM]_2_S_4_O_6_ with respect to [BMIM]_2_S_4_O_6_ (30,329 and 12,051 mPa·s at 20 °C, respectively) is therefore consistent with this general behavior.

**Figure 5:**
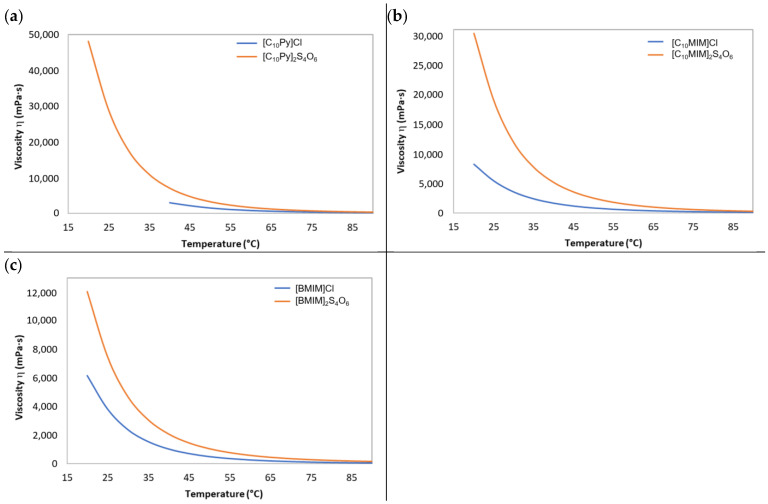
(**a**–**c**): Viscosity (mPa·s) of tetrathionate ILs as a function of temperature compared with their halide precursors.

The viscosity of the synthesized tetrathionate ILs was found to be sensibly higher than the viscosity of their respective halide precursors, as is shown by the plots reported in Figure 5. Even the pyridinium chloride IL (**1a**), solid at room temperature, once melted (40 °C), displayed a lower viscosity compared with the pyridinium tetrathionate IL (**2a**) (Figure 5a). This is not surprising considering that chloride is a monovalent anion, while tetrathionate is a divalent one. The capability of divalent anions to be involved in strong interactions with multiple cations tends to promote the formation of a more solid network of ionic interactions within the ionic liquid with respect to what happens for monovalent anions, hindering the free movement of ions in the liquid and leading to a more viscous behavior [60].

### 2.4. Sulfur Solubilization

**Figure 6:**
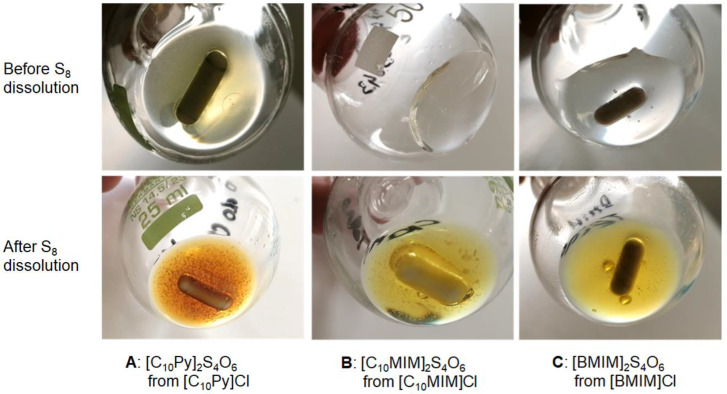
Pictures of ILs before sulfur dissolution (**top**) and after the sulfur dissolution limit was reached, with the visible formation of particles of undissolved sulfur (**bottom**).

In order to evaluate the possible applicability of the presented tetrathionate ILs as viable media for the dissolution of elemental sulfur, S_8_ solubility was determined in each one of the prepared ILs, at various temperatures and in the presence of co-solvents, according to the procedure reported in the Materials and Methods Section. A comparison with the literature solubility data of other sulfur dissolving ILs and organic solvents is also provided. The maximum solubility limit could be easily determined by visual inspection, since the undissolved sulfur powder aggregates in granules, which are clearly visible, as is possible to appreciate in Figure 6. The results of the solubility tests are summarized in Table 3.

The performed sulfur solubility tests displayed that both the pyridinium and the imidazolium long chain tetrathionate ILs could dissolve up to 14 wt% of elemental sulfur at 120 °C, with the solubility noticeably increasing at higher temperatures. The two ILs display almost substantially identical sulfur dissolution profiles. Unexpectedly, [BMIM]_2_S_4_O_6_, which is the IL presenting the lower viscosity, was found to provide worse sulfur dissolution performances. It can be concluded that viscosity does not represent a determining parameter in the dissolution of elemental sulfur in ionic liquids, different from what was reported in the literature about the dissolution of other compounds, such as polymers [61]. Comparing the obtained results with the literature data, it can be observed that the tetrathionate anion imparts a much higher sulfur solubility to the ILs with respect to bistriflimide and tosylate anions. Chen et al. and Boros et al. reported sulfur solubility values in 1-ethyl-3-methylimidazolium and 1-ottyl-3-methylimidazolium bistriflimide and in triisobutilmethylphophonium and 1-hexyl-1-methylimidazolium tosylate to not exceed 2 wt% at temperatures up to 125 °C [62,63]. On the other hand, Boros et al. reported, in the same work, a panel of highly performing sulfur solubilizing ILs featuring [BMIM]^+^ cations and *N,N*-dialkyldithiocarbamate, alkyltrithiocarbonate, and *O*-alkyldithiocarbonate anions capable of solubilizing more than 20 wt% of sulfur at 125 °C. Despite these remarkable solvent capabilities, thiocarbamate and thiocarbonate systems cannot be directly prepared from HydroClaus process byproducts (as it so happens for the tetrathionate ILs), meaning that they do not offer the same economical and procedural advantages. Furthermore, the preparation of dithiocarbamate, trithiocarbonate, and dithiocarbonate systems involves the use of the dangerous and neurotoxic CS_2_ precursor, which makes these ILs a severely lesser green option with respect to polysulfide ones [63,64,65].

**Figure 7:**
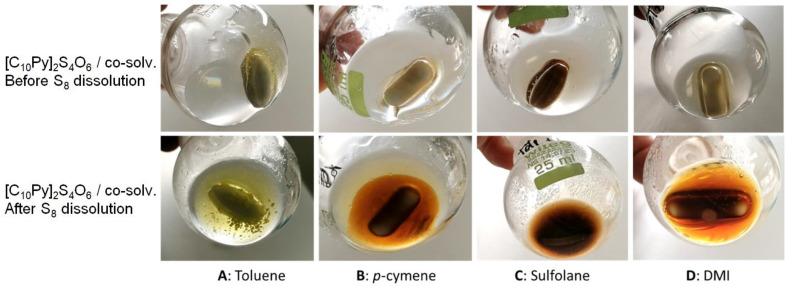
Images of IL/co-solvent system before sulfur dissolution (**top**) and after the sulfur dissolution limit was reached, with the visible formation of particles of undissolved sulfur (**bottom**).

A second set of solubility tests was carried out with IL/ co-solvent systems (Table 4). The sulfur solubility was also determined in the corresponding pure organic solvent as a matter of comparison (Table 4). The aim of these tests was to reduce the cost of the solvent system by mixing the IL with less expensive organic solvents. Considering the good sulfur dissolution properties exhibited in the previous test, [C_10_Py]_2_S_4_O_6_ was selected as the IL component of the mixture, while a panel of high boiling organic solvents, toluene, p-cymene, sulfolane, and DMI, were investigated as possible co-solvents. Toluene was selected as the reference solvent for sulfur dissolution. The other solvents are specifically chosen as follows: p-cymene is a renewable alternative to toluene; sulfolane and DMI are widely used industrial solvents with a lower flammability than toluene. Indeed, while the flash point of toluene is 4 °C, those of sulfolane and DMI are 177 °C and 120 °C (open cup), respectively. These last two values match the temperatures of the dissolution process. The test was performed adopting a 1:1 (wt/wt) IL/co-solvent ratio for the mixtures. Despite the IL [C_10_Py]_2_S_4_O_6_ resulting in being not miscible with toluene and *p*-cymene, these mixtures were studied anyway, since it was still be possible that the sulfur dissolution would create a homogeneous tertiary system. However, a slight second phase was still observed even after the addition of sulfur. The results obtained are shown in Table 4 and Figure 7.

The best solubility results were obtained with the [C_10_Py]_2_S_4_O_6_/DMI (1:1) mixture, which exhibited a sulfur solubilization up to 12 wt%, while the mixture [C_10_Py]_2_S_4_O_6_/sulfolane displayed the worst results, achieving only 4 wt%. The mixtures containing toluene and *p*-cymene manifested intermediate behavior, respectively, dissolving sulfur up to 8 wt% and 10 wt%.

By comparing the obtained results with the solubility of elemental sulfur in the pure organic solvents, the result obtained with DMI is particularly noteworthy. For toluene and *p*-cymene, the solubility results obtained for the pure solvents are much higher (especially for toluene) than what were observed for the IL mixtures and, interestingly, the performances displayed by the mixture were also lower than the solubility values obtained for the pure ionic liquid. It can be concluded that the presence of [C_10_Py]_2_S_4_O_6_ exhibits a strong sulfur-related antisolvent effect on toluene and *p*-cymene (and vice versa), whose intensity is quite surprising in consideration of the minimal solubility of the IL in these solvents, as evidenced by the formation of the double phase. The mixture of [C_10_Py]_2_S_4_O_6_ and sulfolane, on the other hand, yielded a system providing a lower solubilization than the pure ionic liquid but higher than the pure sulfolane. Nevertheless, also in this case, a sulfur antisolvent effect can be appreciated since the expected solubility value in case of no reciprocal influence between the IL and sulfolane (theoretical weighted average) would have been 7.5 wt%, much higher than what was obtained. Finally, the DMI mixture, besides providing the best result, even if slightly lower than the one obtained with pure [C_10_Py]_2_S_4_O_6_, is the only system where no antisolvent effects took place and, conversely, a weak cooperative effect can be appreciated. In this case, a 10 wt% would have been expected if the IL and solvent exerted no reciprocal influence on the solubilization of sulfur, while the experimental data yielded a 12 wt% value.

## 3. Materials and Methods

1-Methylimidazole, 1-chlorobutane, and *p*-cymene were purchased from Alfa Aesar and vacuum distilled before the use. Ethanol (EtOH), iodine (I_2_), diethylether (Et_2_O), chloroform (CHCl_3_), and acetonitrile (CH_3_CN) were purchased from Merk and used as received. Pyridine, sodium thiosulfate pentahydrate (Na_2_S_2_O_3_•H_2_O), 1-chlorodecane, sulfolane, and dimethyl imidazolidinone (DMI) were obtained from Sigma Aldrich, and magnesium sulfate anhydrous (MgSO_4_) from Fluka.

^1^H-NMR spectra were recorded in CD_3_OD with a Bruker AVANCE II operating at 400 MHz. ^13^C NMR spectra were recorded at 100 MHz. The experiments were performed at 25 °C. The following abbreviations are used: s = singlet, d = doublet, t = triplet, q = quartet, m = multiplet, and tt = triple triplet. The chemical shift (δ) references the chemical shift in CD_3_OD (δ_H_ 3.31), and J-values are given in Hz.

ATR-FTIR spectra were recorded with the spectrophotometer from Agilent Technologies, IR Cary 660 FTIR, using a Diamond crystal macro-ATR accessory. The spectra were measured in a range from 4000 to 400 cm^−1^, with 32 scans and a resolution of 4 cm^−1^.

Theoretical calculations to simulate the FTIR spectra of tetrathionate systems were performed with Gaussian 16 software using the B3LYP/6-311++G (2d, 2p) method. The empirical dispersion correction scheme D3 was used. A server based on dual Xeon Gold 5218 with 128 Gb of RAM was used [67,68].

The thermal behavior of the tetrathionate ILs and the related precursors were investigated using a TA Instruments DSC, Q250 (TA Instruments, New Castle, DE, USA). Dry, high purity N_2_ gas with a flow rate of 50 cm^3^ min^−1^ was purged through the sample. In a typical experiment, 3–6 mg of IL was loaded into a pin-holed hermetic aluminum crucible. The analyses were carried out in a temperature range within −90 °C and 100 °C, with a 10 °C min^−1^ scanning rate. The sample was initially cooled down to −90 °C and maintained for 1 min at this temperature. Then, the sample was heated up to 100 °C (first heating run). After 1 min at 100 °C, the sample was cooled down to −90 °C (cooling run) and heated again to 100 °C (second heating run). The thermal stability of the considered tetrathionate ILs and sodium tetrathionate was investigated by thermal gravimetric analysis (TGA), conducted in a TA Instruments Q500 TGA (weighing precision ± 0.01%, sensitivity 0.1 µg, baseline dynamic drift of <50 µg). The Curie point of a nickel standard was used to perform the temperature calibration, while weight standards of 1000, 500, and 100 mg were used for mass calibration. All the standards were supplied by TA Instruments Inc. In total, 15–20 mg of each sample, previously accurately dried under high vacuum at 70 °C overnight, were heated in a platinum crucible as a sample holder. First, the heating mode was set to isothermal at 100 °C under N_2_ (80 cm^3^ min^−1^) for 15 min. Then, the sample was heated from 50 to 700 °C with a heating rate of 10 °C min^−1^ under nitrogen (80 cm^3^ min^−1^) and maintained at 700 °C for 1 min. The mass change was recorded as a function of temperature. During the TGA of sodium tetrathionate, the isothermal stage at 100 °C was skipped.

Mass spectra were recorded in both positive and negative ion mode on a high-resolution mass spectrometer Q Exactive Plus Orbitrap-based FT-MS system, equipped with an ESI source (ThermoFisher Scientific Inc., Bremen, Germany). Samples were dissolved in methanol and injected into the ESI source using a syringe pump (flow rate of 5 µL/min).

The viscosities of the tetrathionate ILs were measured, as a function of temperature, using a modular compact rheometer (MCR 302, Anton Paar, Graz, Austria) equipped with a plate–plate geometry (diameter of 5 cm) and a protective hood. Before conducting the analyses, all the samples were subjected to a pre-shear phase to improve the uniformity and homogeneousness of the samples on the plate. Initially, a flow curve measurement was performed with a shear rate from 0.1 s^−1^ to 100 s^−1^ at 20 °C. A total of 30 data points was collected by the rheometer every 15 s. Then, the effect of temperature was evaluated, performing the analysis in the 20 °C to 90 °C temperature range, applying a constant shear rate within the Newtonian liquid range of the considered ILs (30 s^−1^). The temperature of the instrument was controlled by a water-cooled Peltier system (H-PTD200, Anton Paar). The viscosity analysis of [C_10_Py]Cl was performed starting from 40 °C, as it was in solid state at room temperature.

### 3.1. Synthetic Procedures

#### 3.1.1. Preparation of Sodium Tetrathionate Dihydrate (Na_2_S_4_O_6_•2H_2_O)

A modification of the literature procedure described by Foss and Hordvik has been adopted [69]. In total, 26.280 g (2.0 eq) of Na_2_S_2_O_3_·5H_2_O were dissolved in 20 mL of H_2_O and added to 13.572 g (1.01 eq) of I_2_ suspended in 10 mL of water. The mixture was left under stirring at room temperature until all the iodine was consumed. A slightly brown colored solution was obtained. After 15 min, the solution was cooled down to 0 °C and EtOH was added to favor the crystallization of Na_2_S_4_O_6_•2H_2_O. The solution was left under stirring (crystallization was slow). After 1 h, crystals were recovered by filtering under vacuum, were washed with cold EtOH and Et_2_O (20 mL × 2), and dried under vacuum at 30 °C. The title compound, appearing as white crystals highly soluble in water, was obtained with a yield of 94% (15.226 g). The presence of the two hydration molecules was assessed by TGA (predicted water loss: 11.76%, observed water loss: 10.93%). ESI-MS *m*/*z*: (+) 292.8264 (100%) (calc. [Na_3_S_4_O_6_]^+^: 292.8265), 562.6639 (35%) (calc. [Na_5_(S_4_O_6_)_2_]^+^: 562.6638); (−) 246.8480 (100%) (calc. [NaS_4_O_6_]^−^: 246.8481), 516.6856 (20%) (calc. [Na_3_(S_4_O_6_)_2_]^−^: 516.6854).

#### 3.1.2. Synthesis of Pyridinium and Imidazolium Chloride ILs (Compounds **1a**–**c**)

The synthesis of the chloride ionic liquids considered in this study was performed, with few modifications, following a literature procedure [70]. Briefly, 0.10 mol (1.00 eq) of amine (pyridine or 1-methyl imidazole) was diluted with MeCN (40 mL) and cooled down at 0 °C, and 1.01 eq. of chlorodecane or chlorobutane was diluted with MeCN (20 mL) and slowly added to the amine solution. After the addition, the reaction was heated at 80 °C for two days (butyl alkyl chain) or one week (decyl alkyl chain) and stirred until the amine was consumed (the progressions of the reaction were followed by TLC). MeCN was removed under reduced pressure, and the crude product was washed with Et_2_O in order to remove the excess of alkyl chain. The desired ionic liquids were obtained in excellent yields (95%, 93% and 96% yield, respectively, for compounds **1a**, **1b**, and **1c**), and their structures and purities were confirmed by NMR and FT-IR analysis (Supplementary Materials, Figures S4–S12).

#### 3.1.3. Synthesis of *N*-Decylpyridinium Tetrathionate ([C_10_Py]_2_S_4_O_6_) (**2a**)

A total of 1.550 g (1.01 eq) of freshly prepared Na_2_S_4_O_6_ was dissolved in 5 mL of H_2_O and combined at room temperature with 2.565 g (2.0 eq) of *N*-decylpyridinium chloride [C_10_Py]Cl (**1a**) dissolved in 10 mL of H_2_O. Immediately, it was possible to notice the formation of a water-immiscible phase. The suspension was kept under stirring for 2 h, after which 20 mL of CHCl_3_ were added, and the organic phase was separated; the aqueous phase was further extracted with CHCl_3_ twice again (2 × 20 mL). The collected organic phases were washed with 20 mL of H_2_O, dried over MgSO_4_, and the solvent removed under reduced pressure. The desired IL [C_10_Py]_2_S_4_O_6_ was obtained as a light-yellow, very viscous liquid in 96.2% yield (3.209 g). ^1^H NMR (400 MHz, Methanol-*d*_4_) δ 9.05 (d, 2H), 8.59 (t, 1H), 8.14 (t, 2H), 4.69 (t, 2H), 2.11–1.96 (m, 2H), 1.46–1.15 (m, 14H), 0.88 (t, 3H). ^13^C NMR (101 MHz, MeOD) δ 146.6, 146.1, 129.6, 63.1, 33.0, 32.6, 30.6, 30.5, 30.4, 30.1, 27.2, 23.7, 14.4. ESI-MS m/z: (+) 220.2054 (100%) (calc. [C_10_Py]^+^: 220.2060), 884.4742 (25%) (calc. [(C_10_Py)_3_(S_4_O_6_)]^+^: 884.4769) (−) 444.0644 (calc. [(C_10_Py)S_4_O_6_]^−^: 444.0649), 776.2000 (25%) (calc. [(C_10_Py)_6_(S_4_O_6_)_4_]^2−^: 776.2004), 1108.3346 (30%) (calc. [(C_10_Py)_3_(S_4_O_6_)_2_]^−^: 1108.3358).

#### 3.1.4. Synthesis of 1-Decyl-3-methylimidazolium Tetrathionate ([C_10_MIM]_2_S_4_O_6_) (**2b**)

A total of 1.363 g (1.01 eq) of Na_2_S_4_O_6_·2H_2_O was dissolved in 5 mL of H_2_O and added to 2.282 g (2.0 eq) of 1-decyl-3-methylimidazolium chloride ([C_10_MIM]Cl) (**1b**) dissolved in 10 mL of water. No phase separation was observed. The mixture was kept under stirring for 2 h at room temperature, after which 20 mL of CHCl_3_ were added and the organic phase was separated; the aqueous layer was further extracted 3 times with CHCl_3_ (3 × 15 mL). The collected organic phases were washed with 20 mL of H_2_O, dried over MgSO_4_, and the solvent was removed under reduced pressure. The desired IL ([C_10_MIM]_2_S_4_O_6_ was obtained as a yellow-shaded, transparent viscous liquid (2.840 g, 96.0% yield). ^1^H NMR (400 MHz, Methanol-*d*_4_) δ 7.57 (dd, *J* = 24.0, 2.0 Hz, 2H), 4.24 (t, 2H), 3.95 (s, 3H), 1.90 (p, *J* = 7.4 Hz, 2H), 1.33 (dq, *J* = 26.0, 4.2 Hz, 14H), 0.89 (t, 3H). ^13^C NMR (101 MHz, MeOD) δ 137.0, 123.6, 123.6, 122.2, 122.2, 49.5, 35.3, 31.7, 30.0, 29.3, 29.2, 29.1, 28.9, 26.0, 22.4, 13.1. The intensity of the signal corresponding to the proton Im-H2 is heavily affected by the fast exchange of this proton in methanol-*d*_4_. This behavior is consistent with the literature data [71]. ESI-MS *m*/*z*: (+) 223.2167 (100%) (calc. [C_10_MIM]^+^: 223.2169); (−) 447.0761 (100%) (calc. [C_10_MIM]S_4_O_6_^−^: 447.0758).

#### 3.1.5. Synthesis of 1-Butyl-3-methylimidazolium Tetrathionate ([BMIM]_2_S_4_O_6_) (**2c**)

A total of 0.972 g (1.01 eq) of Na_2_S_4_O_6_ was dissolved in 5 mL of H_2_O and added to 1.098 g (2.0 eq) of 1-butyl-3-methil chloride ([BMIM]Cl) (**1c**) in 10 mL of water at room temperature. No phase separation was observed. After 1 h, the water was evaporated and EtOH (15 mL) was added to precipitate the NaCl and the excess of Na_2_S_4_O_6_. The salts were removed by filtration, EtOH was evaporated, and this precipitation/filtration/drying process was carried out twice more. The desired ionic liquid (BMIM)_2_S_4_O_6_ was obtained as a colorless viscous liquid (1.582 g, 94.5% yield). ^1^H NMR (400 MHz, Methanol-*d*_4_) δ 9.00 (s, 1H), 7.59 (dt, *J* = 23.1, 1.9 Hz, 2H), 4.26 (t, *J* = 7.4 Hz, 2H), 3.96 (s, 3H), 1.99–1.79 (m, 2H), 1.49–1.31 (m, 2H), 0.98 (t, *J* = 7.4 Hz, 3H). ^13^C NMR (101 MHz, MeOD) δ 138.2, 124.9, 123.5, 50.6, 36.6, 33.2, 20.5, 13.8. ESI-MS *m*/*z*: (+) 139.1288 (calc. [BMIM]^+^: 139.1230); (−) 224.8660 (25%) (calc. [HS_4_O_6_]^−^: 224.8661), 362.9821 (100%) (calc. [(BMIM)S_4_O_6_]^−^: 362.9819), 865.0876 (20%) (calc. [(BMIM)_3_(S_4_O_6_)_2_]^−^: 865.0868).

### 3.2. Sulfur Dissolution Procedures

#### 3.2.1. Sulfur Dissolution in Pure IL or Organic Solvent

In a typical experiment, 5.0 g of IL or organic solvent were heated to the selected temperatures (90 °C, 100 °C, 110 °C, and 120 °C). Then, elemental sulfur was added (1 wt%), and the mixture was stirred until sulfur completed dissolution. When the sulfur was completely dissolved, further sulfur was added (0.5–1 wt%) until the solubility limit was reached. The dissolution process was repeated in triplicate for each temperature and each tested tetrathionate IL and organic solvent.

#### 3.2.2. Sulfur Dissolution in IL/Co-Solvent System

The IL chosen for the test was [C_10_Py]_2_S_4_O_6_ and the co-solvents selected were toluene, *p*-cymene, sulfolane, and DMI. In a typical experiment, 5.0 g of IL/ co-solvent mixture 1:1 wt/wt, was heated to 110 °C (in the case of IL/toluene mixture) or 120 °C; then, elemental sulfur was added (1 wt%), and the mixture was stirred until sulfur complete dissolution. After sulfur complete dissolution, more sulfur was added (0.5–1 wt%) until the solubility limit was reached (visual inspection). The dissolution process was repeated in triplicate.

## 4. Conclusions

Within this work, a panel of sulfur-dissolving ionic liquids featuring pyridinium and imidazolium cationic heads and tetrathionate anions was synthesized and characterized, along with the chloride ILs precursors. The synthesis of the tetrathionate ILs presented here has been achieved through a novel synthetic route, firstly reported in this work, which represents a significant improvement with respect to the state-of-the-art Volynskii–Smolyaninov reaction. The first step of the developed procedure allows for an immediate, quantitative, and highly selective preparation of sodium tetrathionate, which is used as starting material during a subsequent ion metathesis step for the efficient preparation of the desired ionic liquid in high purity. The proposed system greatly expands the variability of synthesizable tetrathionate ILs, overcoming all the major issues affecting the Volynskii–Smolyaninov procedure, like the effect of the IL cation on the outcome of the reaction, and the unpredictability of the polythionate species formed during the reaction.

The proof-of-concept of the applicability of the synthesized tetrathionate ILs for the dissolution of elemental sulfur was carried out, providing good preliminary evidence of the suitability of these ILs in this field. From the perspective of circular economy, the potential use of ILs derived from sulfur for dissolving elemental sulfur is undoubtedly advantageous. The obtained results pointed out satisfactory performance, comparable to the ones of toluene, a solvent already well investigated in the literature for this application. However, the utilization of ILs, whether in their pure form or in combination with low-flammable organic solvents, offers a distinct advantage in terms of safety during the dissolution process when compared with the use of toluene. The test of binary mixtures of organic solvents and ILs gave positive results only when DMI was used, while for the other solvents, toluene included, an evident reduction in the sulfur solubility in the mixture with respect to the pure components was observed. The proposed tetrathionate ILs proved overall to be interesting candidates as sulfur solvents, but further investigation on the topic would be needed to assess their fruitful application in the industrial context.

## Figures and Tables

**Table 1:** DSC analysis results of ILs **1a**–**c** and **2a**–**c**. The following abbreviations have been adopted: glass transition temperature (*T*_g_), cold crystallization temperature and enthalpy (*T*_cc_ and ΔH_cc_, respectively), melting temperature and enthalpy (*T*_m_ and ΔH_m_, respectively).

IL	T_g_ (°C)	T_cc_ (°C)	ΔH_cc_ (kJ mol^−1^)	T_m_ (°C)	ΔH_m_ (kJ mol^−1^)
[C_10_Py]Cl (**1a**)	−69.39	−4.43; 22.03	22.897 *	37.79; 53.34	25.834 *
	−59.36				
	−52.85	25.40	2.385	37.54	2.075
[C_10_MIM]Cl (**1b**)	−61.70	−31.20; 0.09	23.595; 2.782	30.10	25.661
	−62.13				
	−51.30				
[BMIM]Cl (**1c**)	−76.50			63.54	11.375
	−62.83				
	−61.88				
[C_10_Py]_2_S_4_O_6_ (**2a**)	−51.08				
	−43.07				
	−38.52				
[C_10_MIM]_2_S_4_O_6_ (**2b**)	−58.91	−6.91	48.624	19.97	49.615
	−58.56				
	−47.24	10.70	3.864	28.82	4.189
[BMIM]_2_S_4_O_6_ (**2c**)	−58.44				
	−49.32				
	−47.38				

Data reported in black refer to the first heating ramp, data reported in blue refer to the subsequent cooling ramp, and data reported in violet refer to the second heating ramp. Multiple data in the same table cell refer to multiple peaks in the same ramp. The thermal phenomena values reported in the text, when not differently specified, refer to the first heating ramp. * This enthalpy value includes multiple peaks.

**Table 2:** TGA results of Na_2_S_4_O_6_ and ILs **1a**–**c** and **2a**–**c**. *T*_5%_ indicates the temperature at which a 5% loss of mass recorded; *T*_onset_ indicates the onset of the Weight% vs. Temperature plot; *T*_peak_ indicates the main degradation peak observed in the dWeight%/dTemperature vs. Temperature plot.

IL	T_5%_ (°C)	T_onset_ (°C)	T_peak_ (°C)
Na_2_S_4_O_6_	187.31	182.98	186.33
[C_10_Py]_2_S_4_O_6_ (**2a**)	196.01	196.96	202.42
[C_10_MIM]_2_S_4_O_6_ (**2b**)	205.84	202.87	209.97
[BMIM]_2_S_4_O_6_ (**2c**)	200.87	200.65	208.39

**Table 3:** Sulfur dissolution capacity of the tetrathionate ILs **2a**–**c**.

	Dissolution Capacity (wt%)			
	Temperature (°C)			
**IL**	**90**	**100**	**110**	**120**
[C_10_Py]_2_S_4_O_6_ (**2a**)	8	10	13	14
[C_10_MIM]_2_S_4_O_6_ (**2b**)	8	10	12	14
[BMIM]_2_S_4_O_6_ (**2c**)	4	6	8	8

**Table 4:** Dissolution of sulfur with IL/co-solvent systems.

IL/Co-Solvent (1:1 wt/wt)	Miscibility	S_8_ Solubility at 120 °C (wt%)	S_8_ Solubility (Pure Solvents) at 120 °C (wt%)
[C_10_Py]_2_S_4_O_6_/toluene	Not miscible	8 *	23 *^,a^
[C_10_Py]_2_S_4_O_6_/*p*-cymene	Not miscible	10	16
[C_10_Py]_2_S_4_O_6_/sulfolane	miscible	4	2
[C_10_Py]_2_S_4_O_6_/ DMI	miscible	12	6

* The test with toluene was carried out at 110 °C, due to the boiling point of this solvent. ^a^ The obtained data is coherent with the literature data (Supplementary Materials) [36,66].

## Data Availability

Data are contained within the article or the Supplementary Materials.

## Supplementary Materials

The following supporting information can be downloaded at https://www.mdpi.com/article/10.3390/ijms26199823/s1.
