# Supplementary material for: A Novel Approach for the Preparation of Tetrathionate Ionic Liquids and Study of Their Sulfur Dissolution Properties
Source: Int J Mol Sci. 2025 Oct 9;26(19):9823. doi: 10.3390/ijms26199823 (PMC12524832; doi:10.3390/ijms26199823)
Supplement: Supplementary file 1 [file ijms-26-09823-s001.zip › ijms-3912735-supplementary.pdf]

A novel approach for the preparation of tetrathionate ionic liquids and study of their sulfur dissolution properties

Supporting Information

Table of contents

ATR-FTIR spectra of $Na_2S_4O_6$ and $Na_2S_2O_3$, and TGA analysis of $Na_2S_4O_6$................. 2

[4]^{1}H-NMR and ^{13}C-NMR of ILs **1** – **6** 4

ATR-FTIR spectra of compounds of $Na_2S_4O_6$, $Na_2S_2O_3$ and of ILs **1** – **6** 7

Characteristic normal modes of the tetrathionate ion system 8

Viscosity of ILs in dependence of temperature 9

TGA of $Na_2S_4O_6$ and of ILs 4 – **6**.. 10

$Na_2S_4O_6$

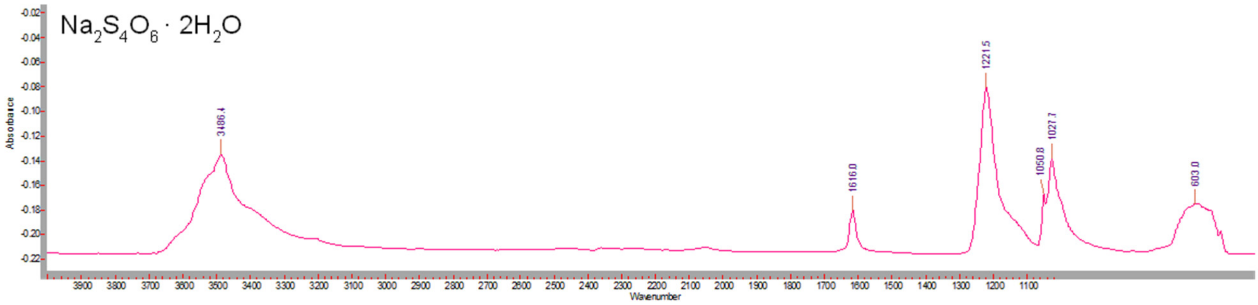

Figure S1. ATR-FTIR of $Na_2S_4O_6$•$2H_2O$

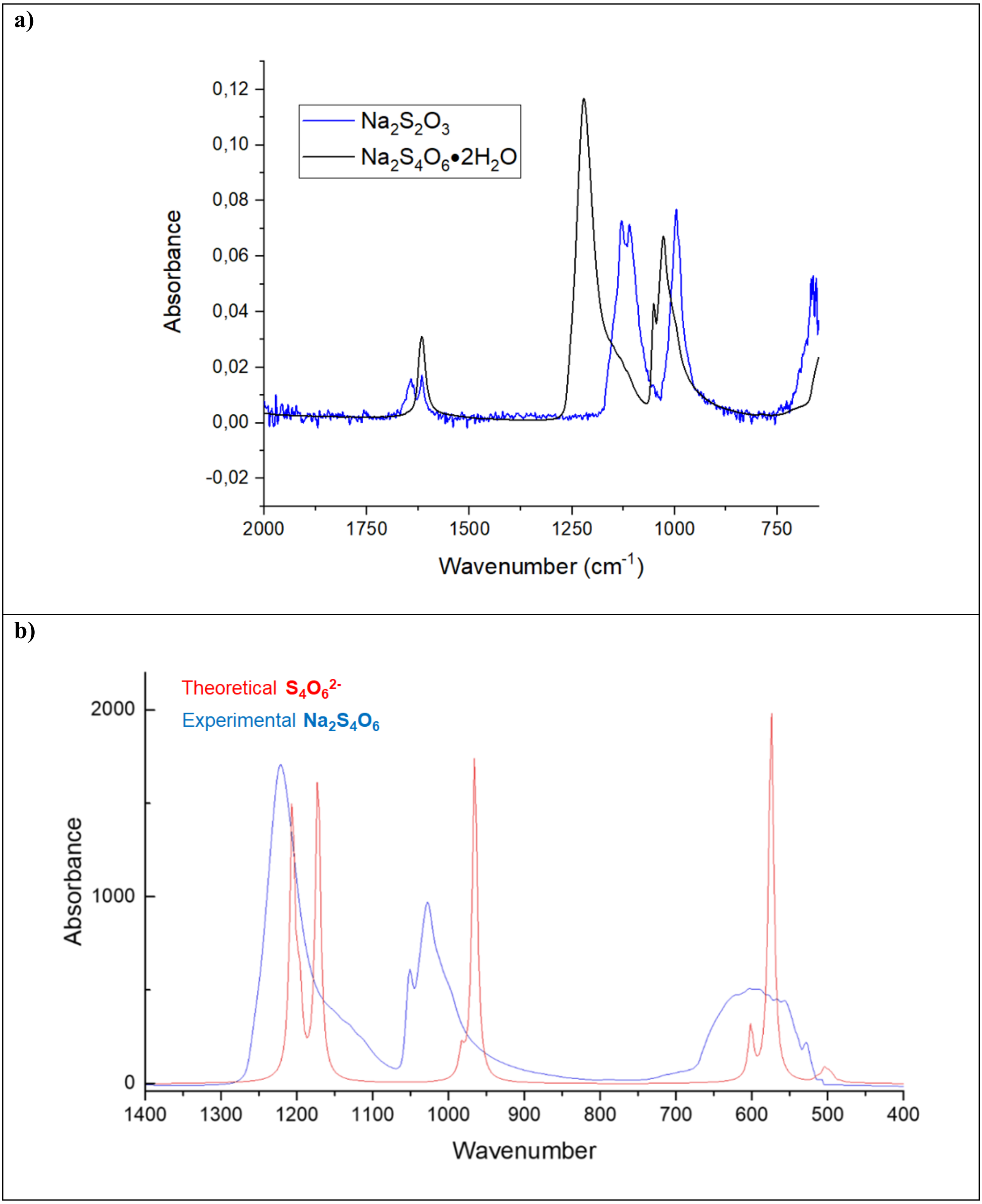

Figure S2. **a)** ATR-FTIR spectra of $Na_2S_4O_6 \bullet 2H_2O$ (black) and $Na_2S_2O_3$ (blue); b) Experimental ATR-FTIR spectra of $Na_2S_4O_6 \bullet 2H_2O$ (blue) and computationally modeled IR spectrum of the $S_4O_6^{2-}$ (red).

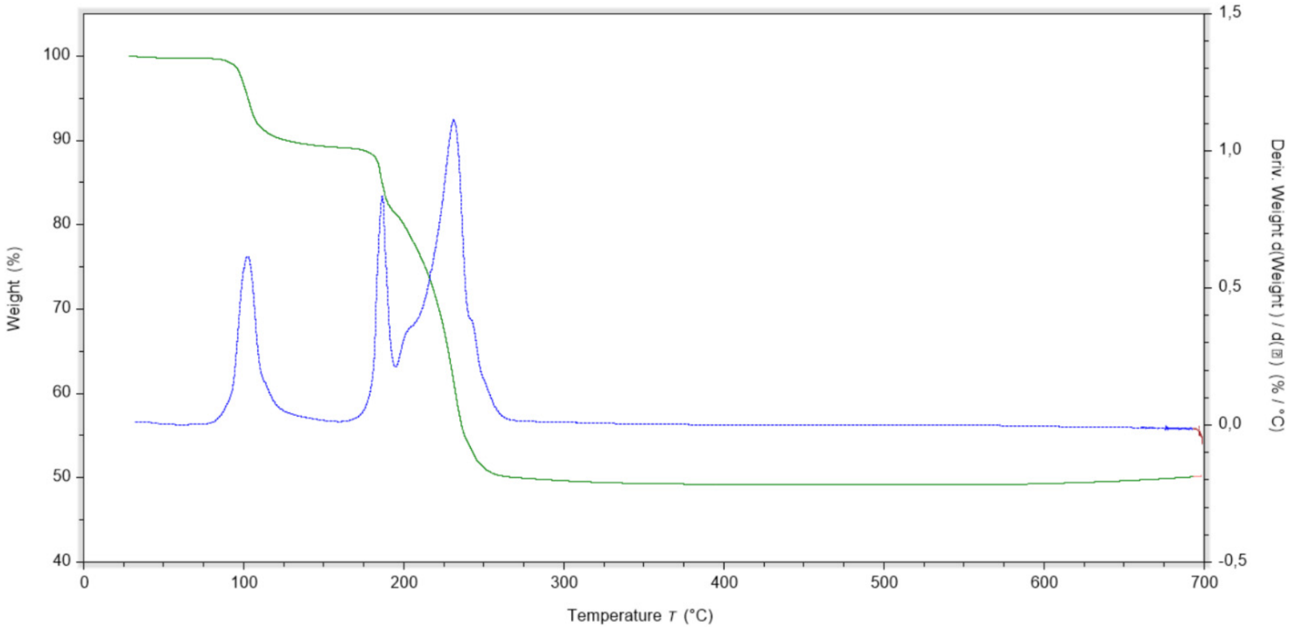

Figure S3. TGA thermogram of compound $Na_2S_4O_6 \bullet 2H_2O$. Weight% vs Temperature plot is reported in green, *d*weight%/*d*Temperature vs Temperature plot is reported in blue.

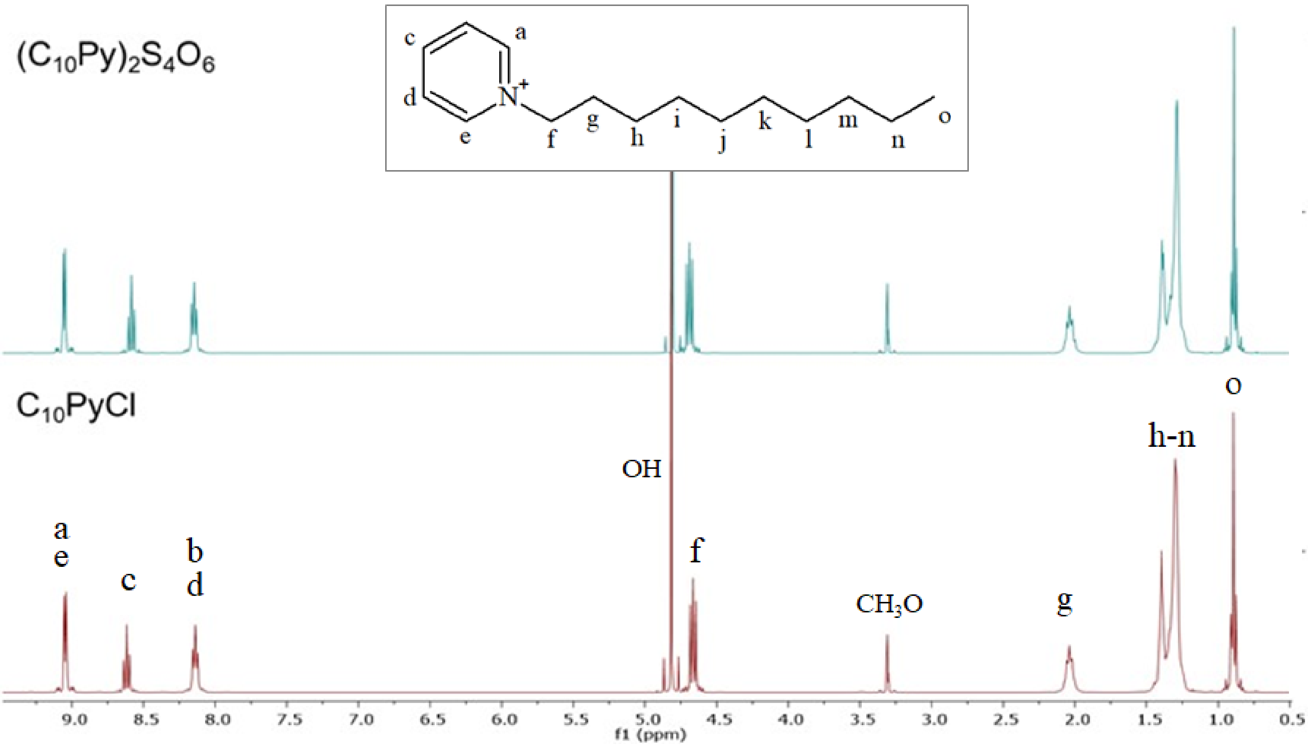

Figure S4. ^{1}H-NMR (in CD_3OD) of compound **4** (top), and **1** (bottom).

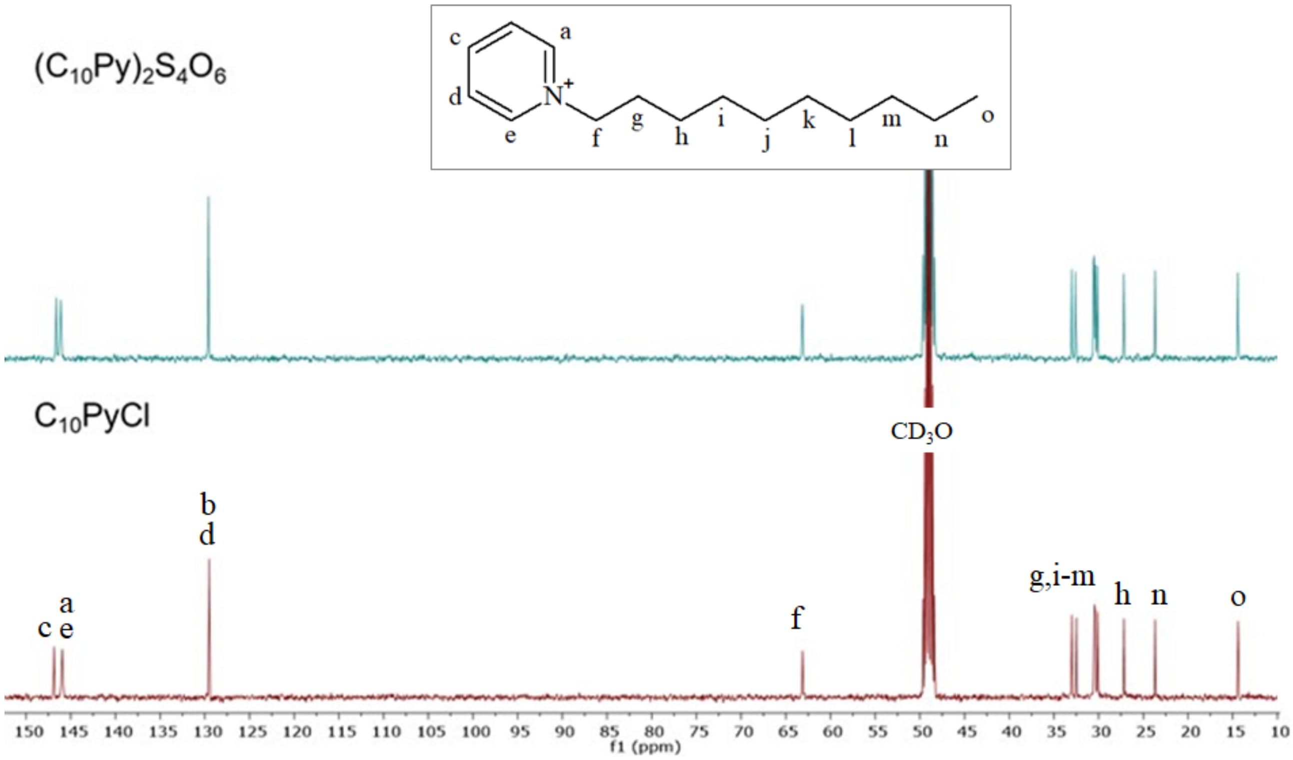

Figure S5. ^{13}C-NMR (in CD_3OD) of compound **4** (top), and **1** (bottom).

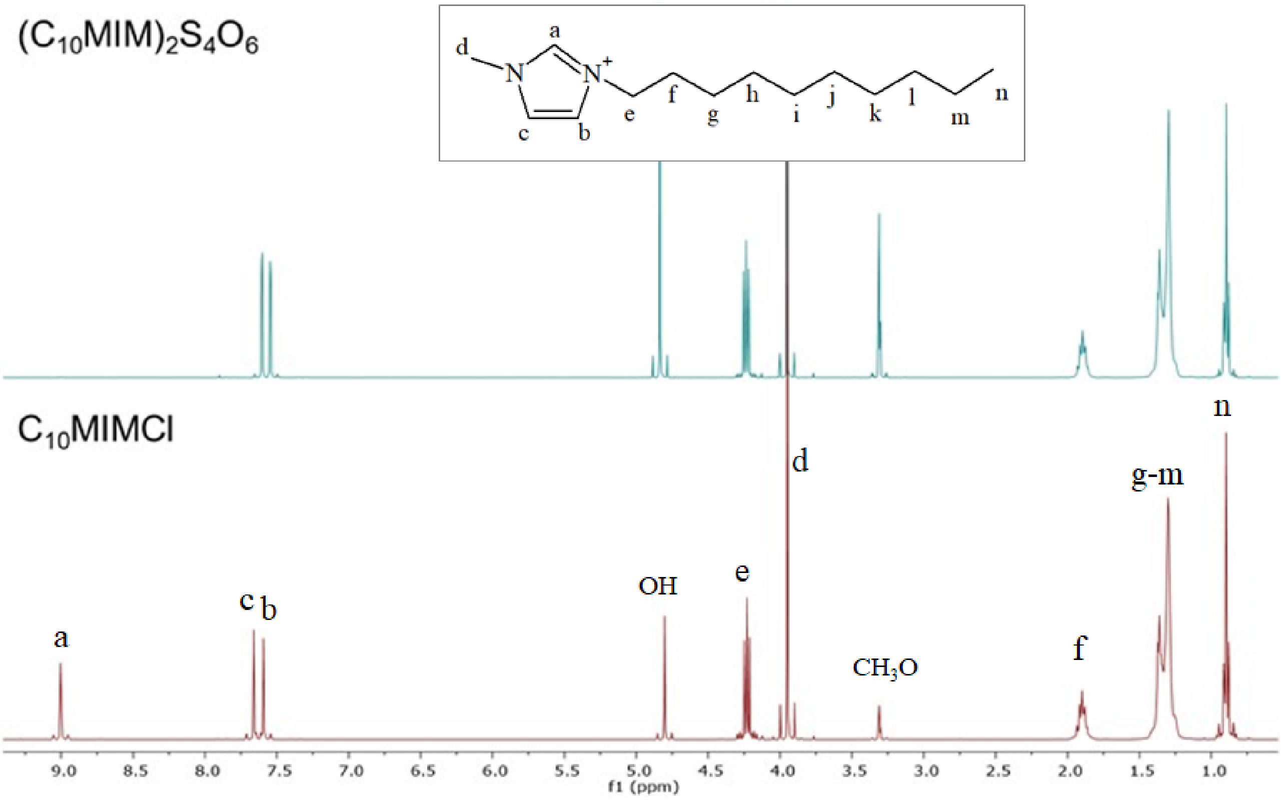

Figure S6. ^{1}H-NMR (in CD_3OD) of compound **5** (top), and **2** (bottom).

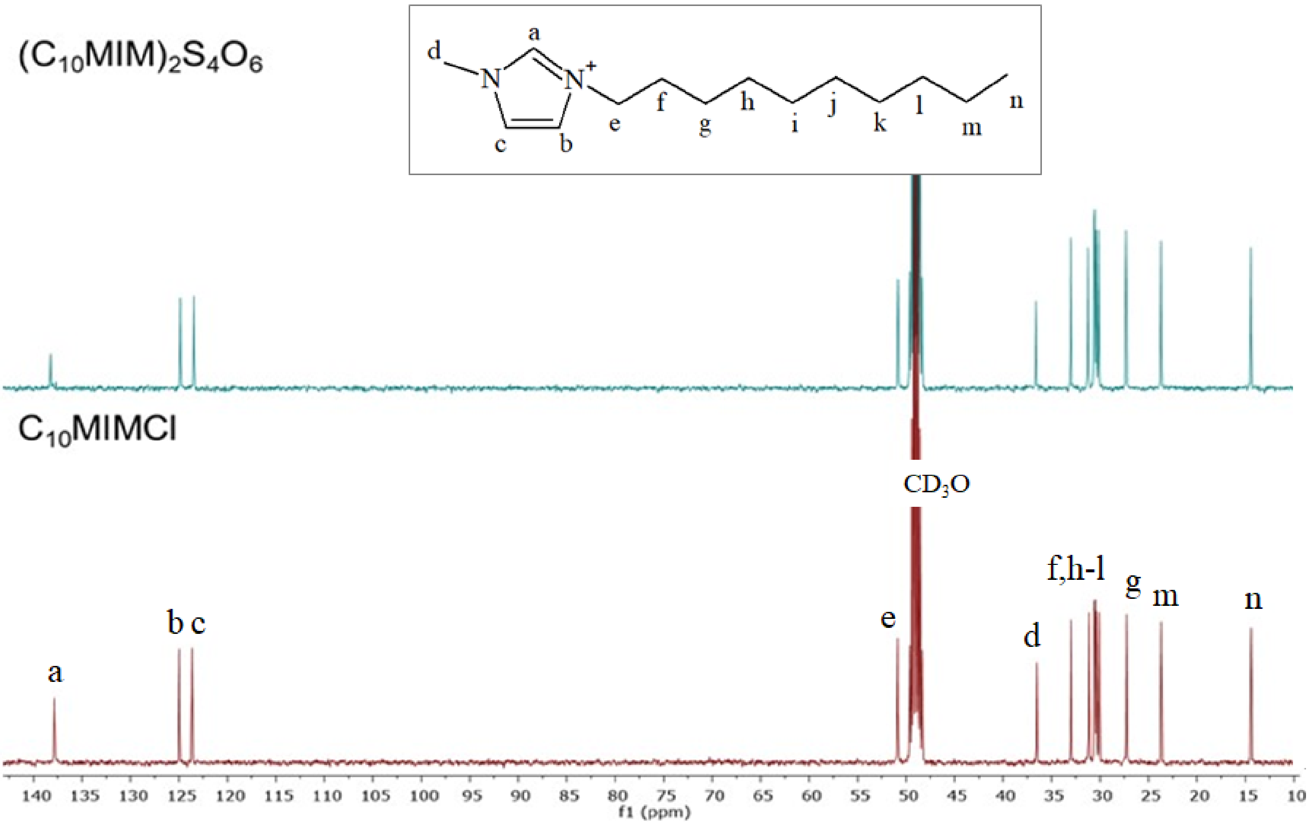

Figure S7. ^{13}C-NMR (in CD3OD) of compound **5** (top), and **2** (bottom).

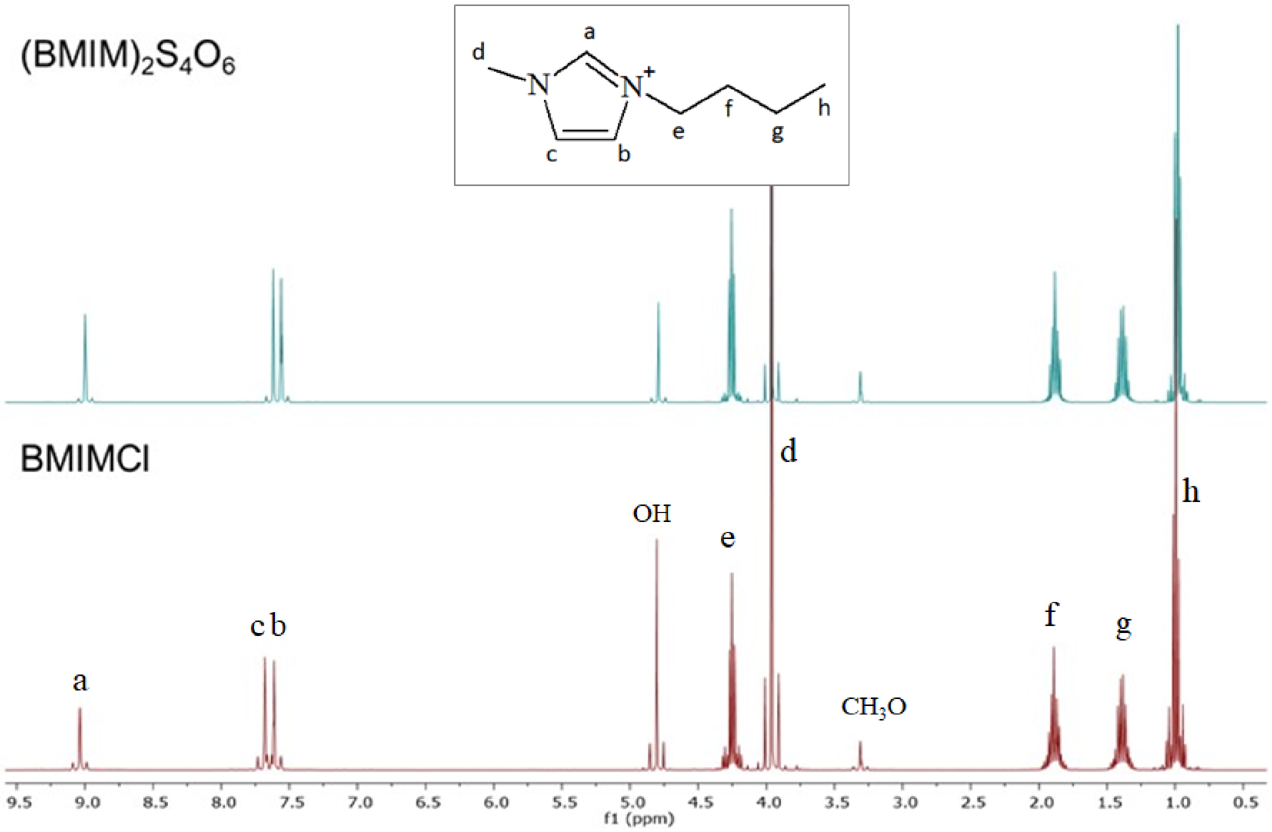

Figure S8. ^{1}H-NMR (in CD3OD) of compound **6** (top), and **3** (bottom).

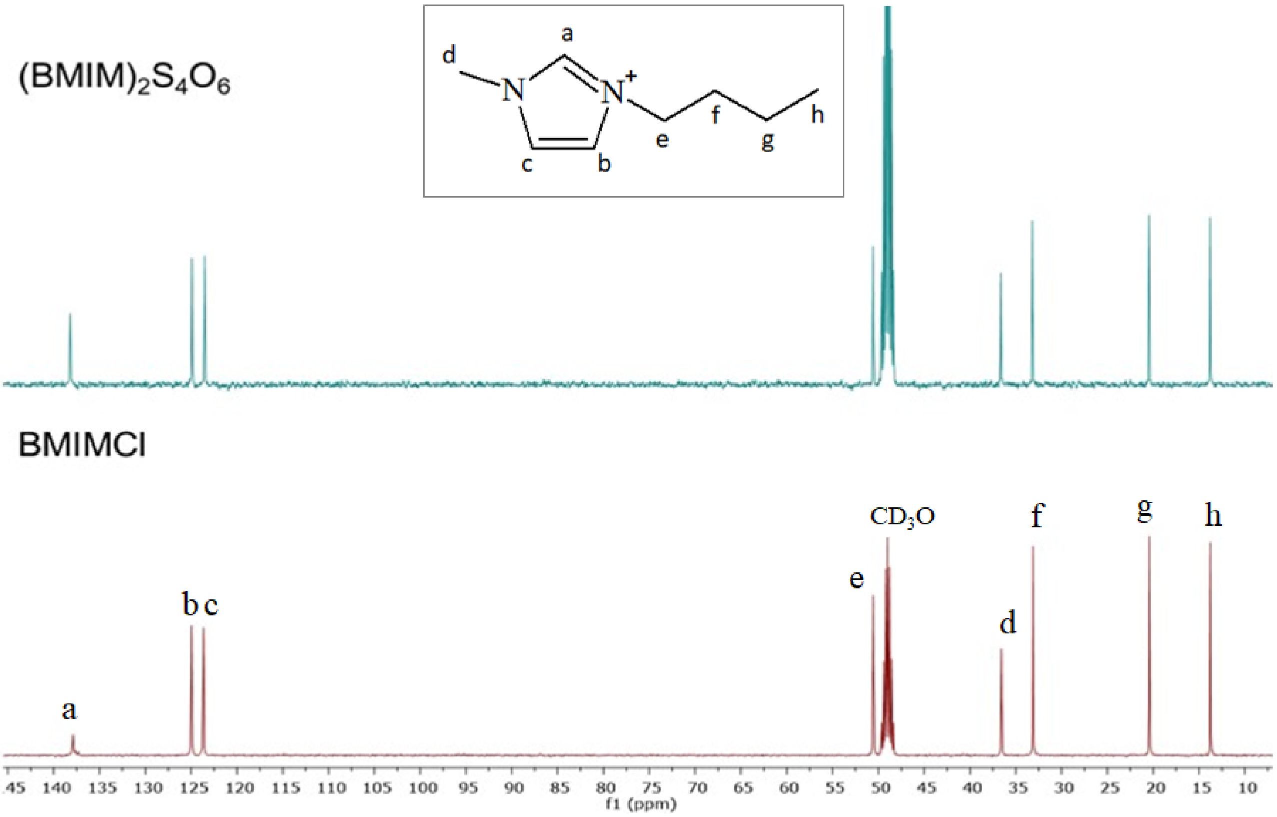

Figure S9. ^{13}C-NMR (in CD3OD) of compound **6** (top), and **3** (bottom).

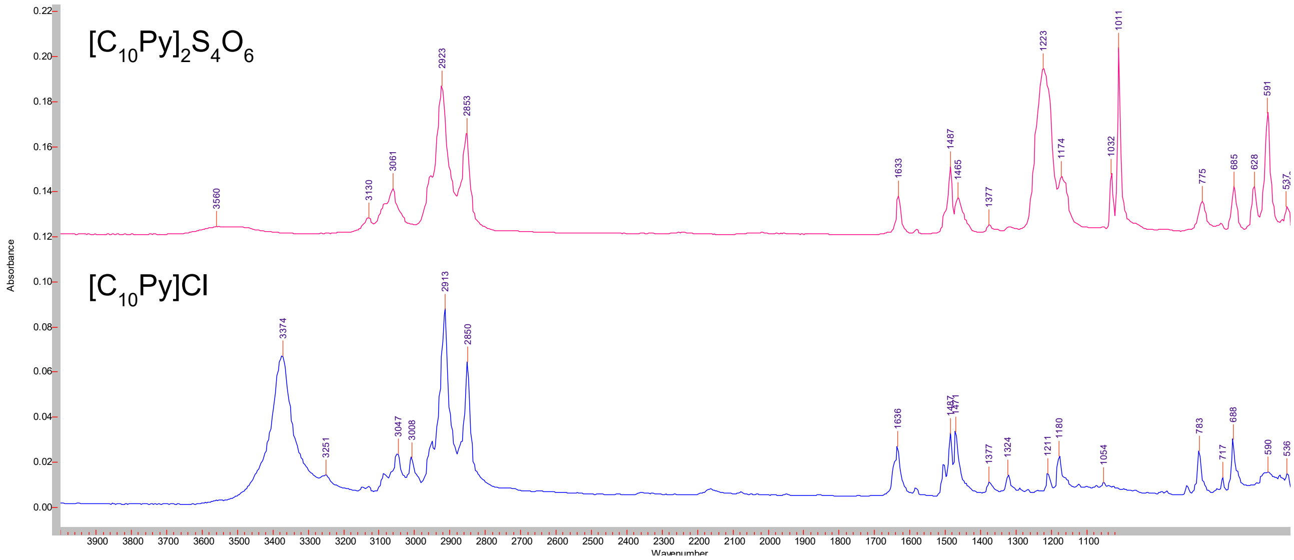

Figure S10. ATR-FTIR of compound **4** (top), and **1** (bottom)

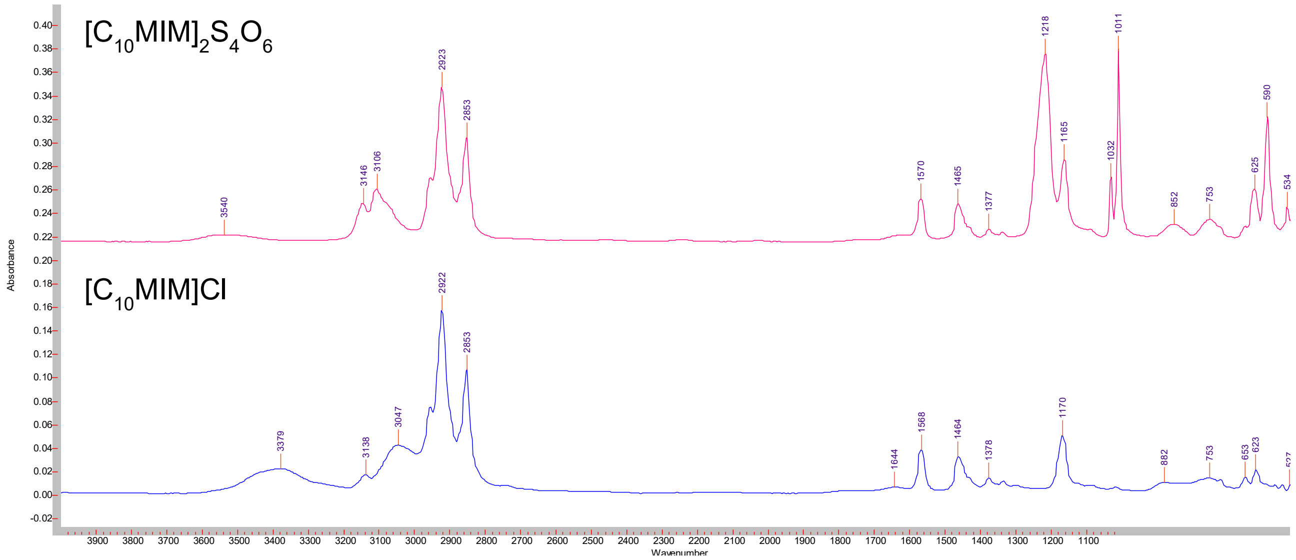

Figure S11. ATR-FTIR of compound **5** (top), and **2** (bottom).

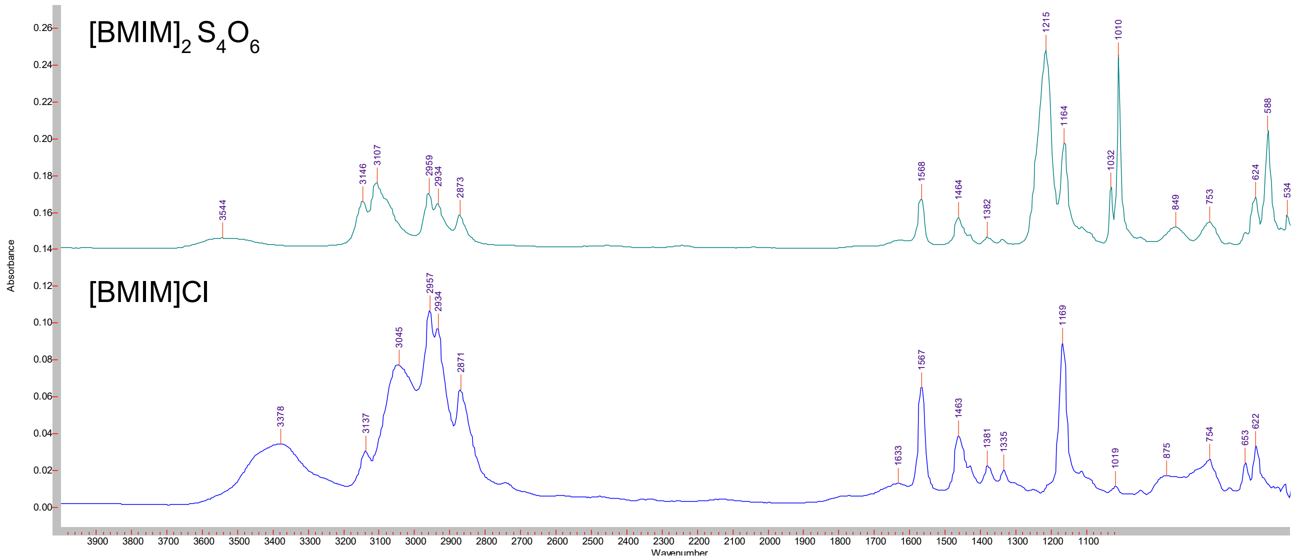

Figure S12. ATR-FTIR of compound **6** (top), and **3** (bottom).

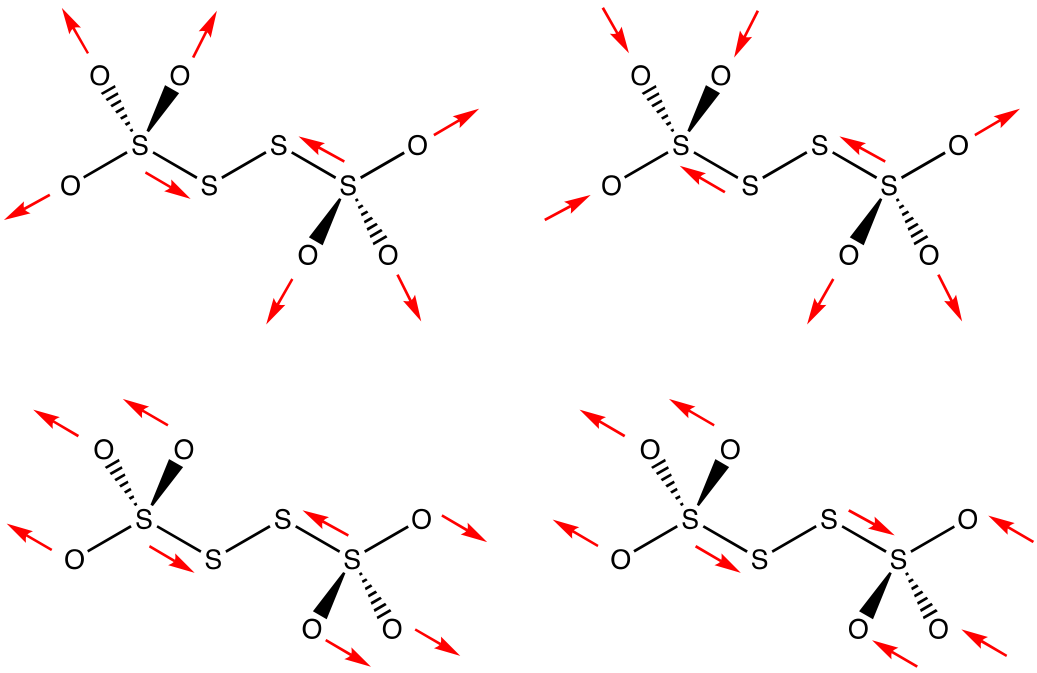

Figure S13. Characteristic normal modes of the tetrathionate ion system. Top: paired symmetric stretching; bottom: paired asymmetric stretching. Left: In phase; right: in counterphase. In the asymmetric stretching the motion of the three oxygen atoms are in counterphase w.r.t. the sulphur one.

Table S1: Dependence of ILs viscosity (mPa·s) on temperature

ILs	Temperature °C														
	**20**	**25**	**30**	**35**	**40**	**45**	**50**	**55**	**60**	**65**	**70**	**75**	**80**	**85**	**90**
**$C_{10}PyCl$**	-	-	-	-	2928	2098	1462	1041	757	561	424	325	253	201	162
**$C_{10}MIMCl$**	8233	5406	3542	2389	1654	1172	846	624	469	359	280	221	177	144	118
**BMIMCl**	6184	3842	2399	1560	1047	727	515	377	281	214	166	130	104	85	70
**$[C_{10}Py]_2S_4O_6$**	48177	28916	17396	10909	7093	4752	3284	2331	1695	1260	956	738	581	464	376
**$[C_{10}MIM]_2S_4O_6$**	30329	18964	11891	7737	5187	3579	2534	1838	1361	1028	789	616	489	393	320
**$[BMIM]_2S_4O_6$**	12051	7488	4697	3077	2089	1465	1058	784	594	459	362	291	237	196	164

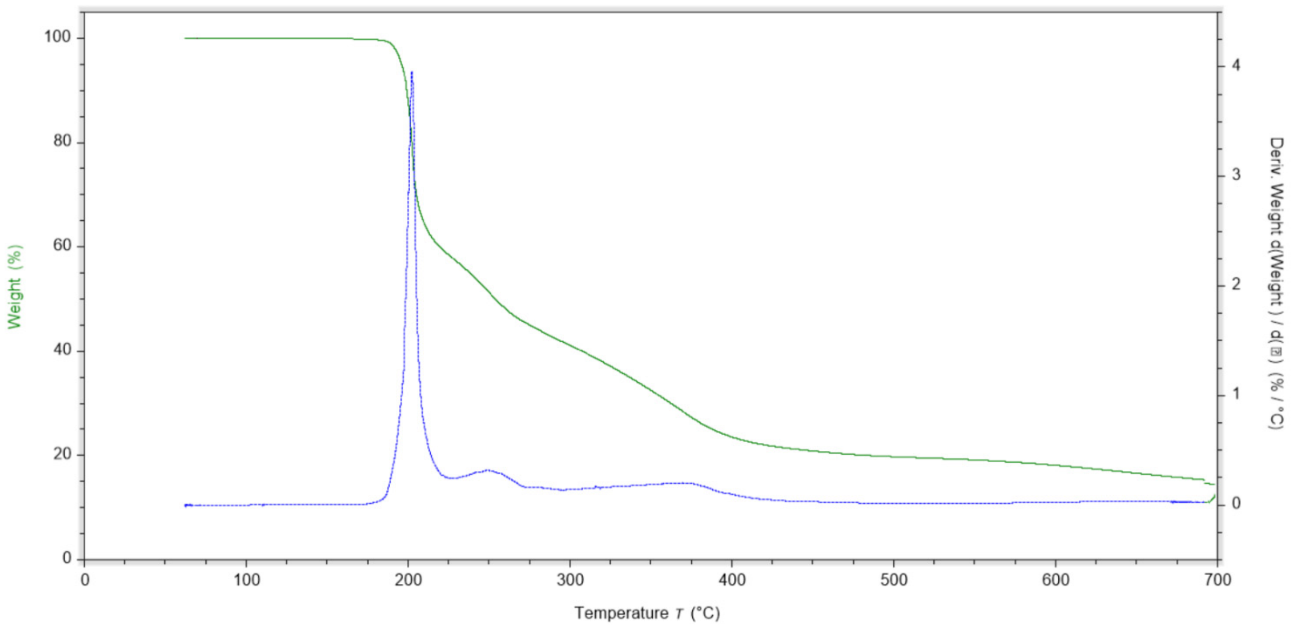

Figure S14. TGA thermogram of compound $[C_{10}Py]_2S_4O_6$. Weight% vs Temperature plot is reported in green, *d*weight%/*d*Temperature vs Temperature plot is reported in blue.

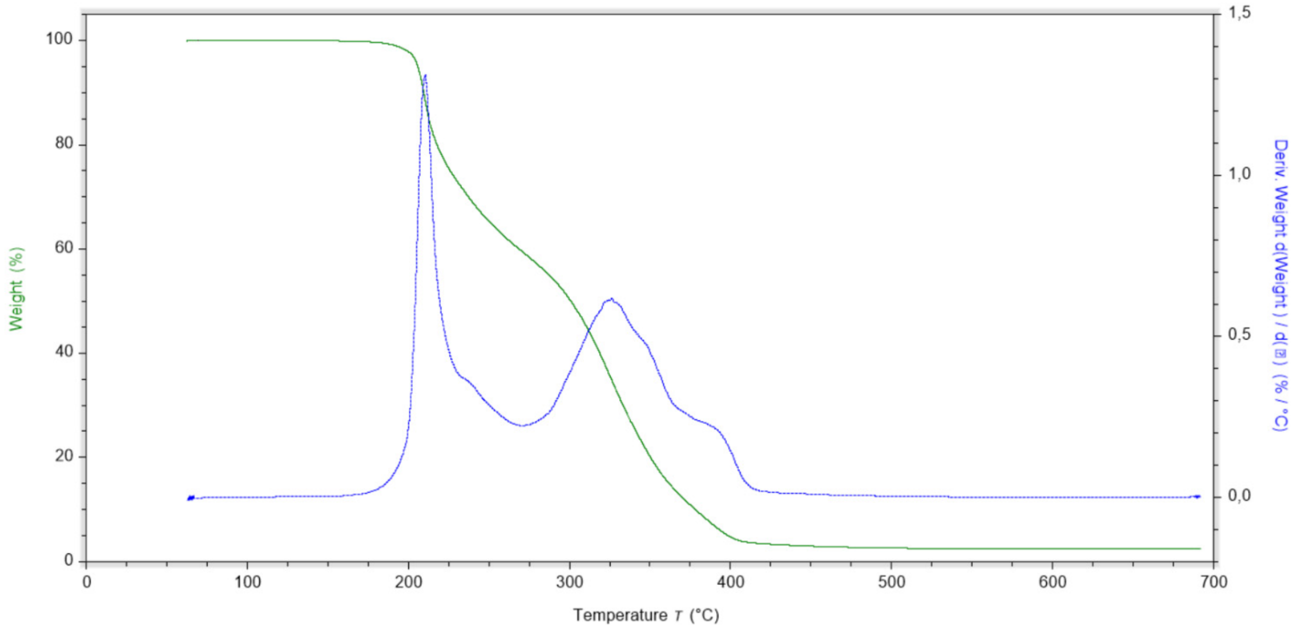

Figure S15. TGA thermogram of compound $[C_{10}MIM]_2S_4O_6$. Weight% vs Temperature plot is reported in green, *d*weight%/*d*Temperature vs Temperature plot is reported in blue.

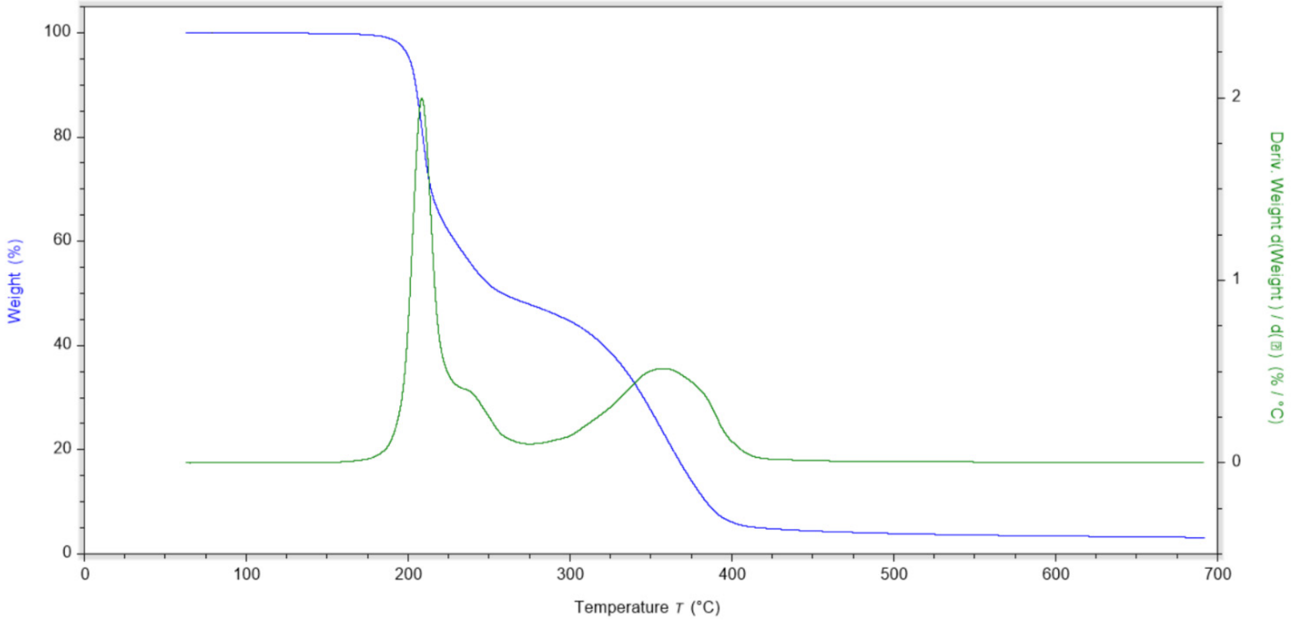

Figure S16. TGA thermogram of compound $[BMIM]_2S_4O_6$. Weight% vs Temperature plot is reported in green, *d*weight%/*d*Temperature vs Temperature plot is reported in blue.
